# Systemic infection facilitates transmission of *Pseudomonas aeruginosa* in mice

**DOI:** 10.1038/s41467-020-14363-4

**Published:** 2020-01-28

**Authors:** Kelly E. R. Bachta, Jonathan P. Allen, Bettina H. Cheung, Cheng-Hsun Chiu, Alan R. Hauser

**Affiliations:** 1 Department of Microbiology-Immunology, Northwestern University, Feinberg School of Medicine, Chicago, IL USA; 2 Department of Medicine, Northwestern University, Feinberg School of Medicine, Chicago, IL USA; 3 Division of Pediatric Infectious Diseases, Department of Pediatrics, Chang Gung Children’s Hospital, Chang Gung University College of Medicine, Taoyuan, Taiwan; 4 Present Address: Department of Microbiology and Immunology, Loyola University Chicago, Stritch School of Medicine, Maywood, IL USA

**Keywords:** Bacterial pathogenesis, Bacteriology, Pathogens

## Abstract

Health care-associated infections such as *Pseudomonas aeruginosa* bacteremia pose a major clinical risk for hospitalized patients. However, these systemic infections are presumed to be a “dead-end” for *P. aeruginosa* and to have no impact on transmission. Here, we use a mouse infection model to show that *P. aeruginosa* can spread from the bloodstream to the gallbladder, where it replicates to extremely high numbers. Bacteria in the gallbladder can then seed the intestines and feces, leading to transmission to uninfected cage-mate mice. Our work shows that the gallbladder is crucial for spread of *P. aeruginosa* from the bloodstream to the feces during bacteremia, a process that promotes transmission in this experimental system. Further research is needed to test to what extent these findings are relevant to infections in patients.

## Abstract (web summary)

*Pseudomonas aeruginosa* bacteremia is presumed to be a ‘dead-end’ for the pathogen and to have no impact on transmission. Here the authors show, in mice, that the bacteria can spread from the bloodstream via the gallbladder to the intestines and feces, leading to transmission to uninfected animals.

## Introduction

Annually in the United States, 440,000 adults contract a health care-associated infection (HAI) resulting in increased mortality for individual patients and an estimated $10 billion in health care costs^1^. While progress has been made in minimizing patient-to-patient spread of HAI bacteria, much remains unknown regarding how these pathogens are transmitted between patients. *Pseudomonas aeruginosa* (PA) is a common cause of HAIs, and in a head-to-head comparison of bloodstream infections (bacteremias), PA was associated with higher mortality than other bacteria^2^. The reasons for this are poorly understood and this, coupled with the rapid emergence of multidrug-resistant (MDR) and extensively drug-resistant PA, make prevention of PA transmission critical^3,4^. However, such prevention has been hampered by an incomplete understanding of in-host PA infection dynamics and their relationship to transmission among hospitalized patients.

Clinically, PA bloodstream infections are regarded as “dead-ends”, meaning that the bacteria in the blood are neither transmitted to other patients nor spread back to the environment. Once bacteria reach the bloodstream, they are either cleared by the host with the help of antibiotics or die with the host if the infection is fatal. In either case, genetic adaptations in PA that confer an enhanced ability to disseminate to the bloodstream are subsequently eliminated from the gene pool. In this regard, PA is thought to differ from gastrointestinal (GI) pathogens, such as *Salmonella enterica* serovar Typhi^5–7^ and *Listeria monocytogenes*^8–10^, which have evolved complex lifecycles that include trafficking from the blood to the gallbladder (GB) and subsequently to the intestines, from which they are excreted in feces to facilitate transmission to new hosts. In contrast, PA bloodstream infections would appear to be accidents from which the bacteria gain no benefit. Thus, the fitness pressures responsible for the evolution of virulence factors that facilitate dissemination of PA to the bloodstream are poorly understood.

To address these issues, we examined the fate of PA bacteria in the bloodstream using a murine model in conjunction with sequence tag-based analysis of microbial populations (STAMP)^9,11^. STAMP utilizes barcoded wild-type strains in combination with deep sequencing to track populations of bacteria, assess infection bottlenecks, and allow for assessment of relatedness of pathogen populations at different sampling sites. We demonstrated that bloodstream PA bacteria seeded the liver and subsequently the GB, where they unexpectedly replicated to extremely high numbers. The bacterial population from the GB seeded the intestines and was excreted in the feces in a manner that facilitated transmission to cage-mate mice. Mice lacking a GB (cholecystectomized) excreted dramatically fewer PA bacteria in their intestinal tracks than wild-type mice, highlighting a critical role for this organ in excretion and environmental contamination. The liver–GB–intestinal excretion pathway may therefore facilitate spread of PA from the bloodstream to the intestines and simultaneously allow amplification of PA. In this way, PA from systemic infections are returned to the environment in high numbers in order to enhance transmission between mice.

## Results

### PA disseminates to the GB following bacteremia

**Fig. 1:**
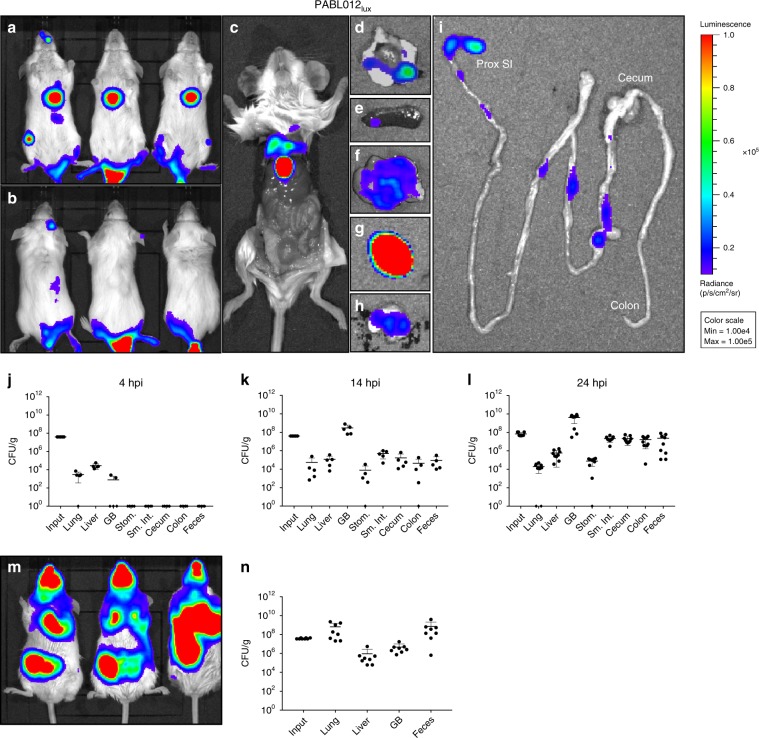
*P. aeruginosa* disseminates to the gallbladder and intestines following bacteremia and pneumonia. Mice (*n* = 3, representative replicate) were intravenously injected with ~2 × 10^6^ CFU of a luciferase expressing strain of *P. aeruginosa*, PABL012_lux_. Ventral (**a**) and dorsal (**b**) views of live mice at 24 hour post infection (hpi) were obtained using IVIS. Mice were subsequently euthanized, the abdominal wall dissected away and internal organs visualized (**c**). The heart and lung (**d**), spleen (**e**), liver (**f**), gallbladder (**g**), stomach (**h**), and remaining intestinal tract (**i**) of a single mouse were separately removed and imaged to visualize bacterial localization. (Since bioluminescence is dependent on oxygen, the signal present in the anaerobic portions of the intestinal tract may underestimate bacterial numbers.) The scale (red = high, blue = low) for all images represents radiance (photons per sec per cm^2^ per steradian) with minimum and maximum values normalized to 1 × 10^4^ and 1 × 10^5^, respectively. Intravenously infected mice housed on standard bedding were euthanized at 4 hpi (**j**, *n* = 5), 14 hpi (**k**, *n* = 5), or 24 hpi (**l**, *n* = 3 × 3 replicates). Bacteria were enumerated from several anatomical sites by plating serial dilutions of organ homogenates. **m** BALB/c mice (*n* = 3, representative replicate) were infected with PABL012_lux_ by intranasal inoculation of ~2 × 10^6^ CFU and imaged at 24 hpi using IVIS. **n** Mice (*n* = 4 × 2 replicates) were subsequently euthanized and bacteria were enumerated from several anatomical sites. Density (CFU per gram [CFU/g] of organ weight) of recovered bacteria are presented (black circles). Organs where no bacteria were recovered are denoted with a diamond on the *x*-axis. Geometric means (horizontal lines) and SD (whiskers) are shown. ProxSI (proximal small intestine), GB (gallbladder), Stom. (stomach), Sm.Int. (small intestine).

We examined the fate of PA during a bloodstream infection utilizing a mouse model in which mice naïve to antibiotic treatment (wild type) were infected via tail vein to allow for consistent and reproducible delivery of bacteria directly into the bloodstream. To define the pattern of dissemination following PA bloodstream infection, we engineered PA strain PABL012 (PABL012_lux_) for in vivo bioluminescence imaging. Mice infected with PABL012_lux_ by tail-vein injection displayed an intense bioluminescent focus in their ventral midsection by 24 hours post infection (hpi) (Fig. 1a, b), which was localized to the GB (Fig. 1c, g). Moderate bioluminescence signals were observed in the stomach (Fig. 1h) and liver (Fig. 1f), while minor signals were detected in the lungs (Fig. 1d), spleen (Fig. 1e), and intestinal tract (Fig. 1i).

Given that quantifying bacterial population sizes from in vivo bioluminescence is imprecise, additional studies were performed to directly enumerate bacterial counts from infected organs. At 4 hpi, PA was detected in the liver, lungs, and a minor proportion of GBs, but no bacteria were detected in the intestinal tract (Fig. 1j). This was followed by a drastic six-logarithm expansion of bacteria in the GB by 14 hpi and similar four- to six-logarithm expansion in the intestinal tract (Fig. 1k). In contrast, total bacterial counts in the lung and liver rose by only one- to two-logarithms over the same period, demonstrating that high levels of PA replication was not a universal phenomenon. Bacterial recovery in the GB reached higher numbers than were originally in the inoculum, suggesting that the GB may be a hospitable niche for PA replication. In support of this, we also observed that PA growth in ex vivo bile preparations approached that of enriched medium (Supplementary Fig. 1). By 24 hpi, this dramatic expansion of bacteria had plateaued with only slight additional increases in all organs (Fig. 1l). Lumenal contents of the intestinal tract contained similar bacterial CFU as observed in whole intestine homogenates (Supplementary Fig. 2g; compare to Fig. 1l), indicating that most of the PA bacteria were in the lumen rather than in the bowel wall. These findings indicate that bacteremia is followed by a dramatic expansion of the bacterial population in the GB and intestines, which is accompanied by PA excretion in the feces.

To determine whether fecal shedding was unique to PABL012, murine gender or genetic background, we tested an additional panel of non-clonal isolates, including the commonly used laboratory strains PAO1 and PA14, the MDR clinical isolate PABL046, as well as 11 additional clinical isolates (Supplementary Table 1). All PA strains shared the same pattern of dissemination to the GB and excretion into the feces (Supplementary Figs. 2a–d and 3) despite being phylogenetically distinct, globally distributed, and representing nine different multilocus sequence types (STs) (Supplementary Fig. 3, Supplementary Table 1). We also challenged male BALB/c mice (Supplementary Fig. 2e) to explore possible gender bias and female C57/BL6 mice (Supplementary Fig. 2f) as an alternate mouse strain background. Male BALB/c mice and female C57/BL6 mice both revealed similar patterns of dissemination, GB expansion, and fecal excretion to the female BALB/c mice. These findings suggest that trafficking to and expansion in the GB, transit to the intestines, and excretion in feces may be universal features of PA bacteremia in mice.

### The excreted PA population originates in the GB

The above data indicated that significant PA replication occurs in the GB, but questions remained as to how systemic PA gained access to the GB and if any population bottlenecks existed during this process. To investigate these questions, we utilized a technique to genetically track bacterial populations referred to as STAMP^11^. We first generated a library of barcoded but otherwise isogenic PABL012 (PABL012_pool_) containing ~4000 unique short (~30 bp) sequence tags inserted into a neutral site on the chromosome. Our goal was to use this library to mathematically estimate the founding population size (*N*_*b*_) at a given sampling site by measuring barcode frequencies from bacterial populations at that site. The *N*_*b*_ value estimates population diversity and reflects how host barriers may shape bacterial populations during systemic infection. Next, we performed in vitro experiments to optimize STAMP. Control experiments demonstrated that barcodes did not influence growth rate (Supplementary Fig. 4a) and were stable in the absence of antibiotic selection (Supplementary Fig. 4b). Using the barcode frequencies of PABL012_pool_, we assessed the accuracy of mathematically calculated founding population size (*N*_*b*_) versus empirically determined *N*_*b*_ values (CFU) from the same samples under controlled in vitro conditions. To accomplish this, we artificially simulated in vitro bottlenecks by sampling serial tenfold dilutions of the PABL012_pool_ and measured the *N*_*b*_ of these samples using both bacterial enumeration and STAMP (Supplementary Fig. 4c). The calculated founding population size underestimated the actual founding population size in vitro, so a calibration curve was generated to adjust our in vivo experimental results to account for this mathematical underestimation, yielding a corrected value referred to as *N*_*b*_ʹ (see Methods section for details)^11^.

**Fig. 2:**
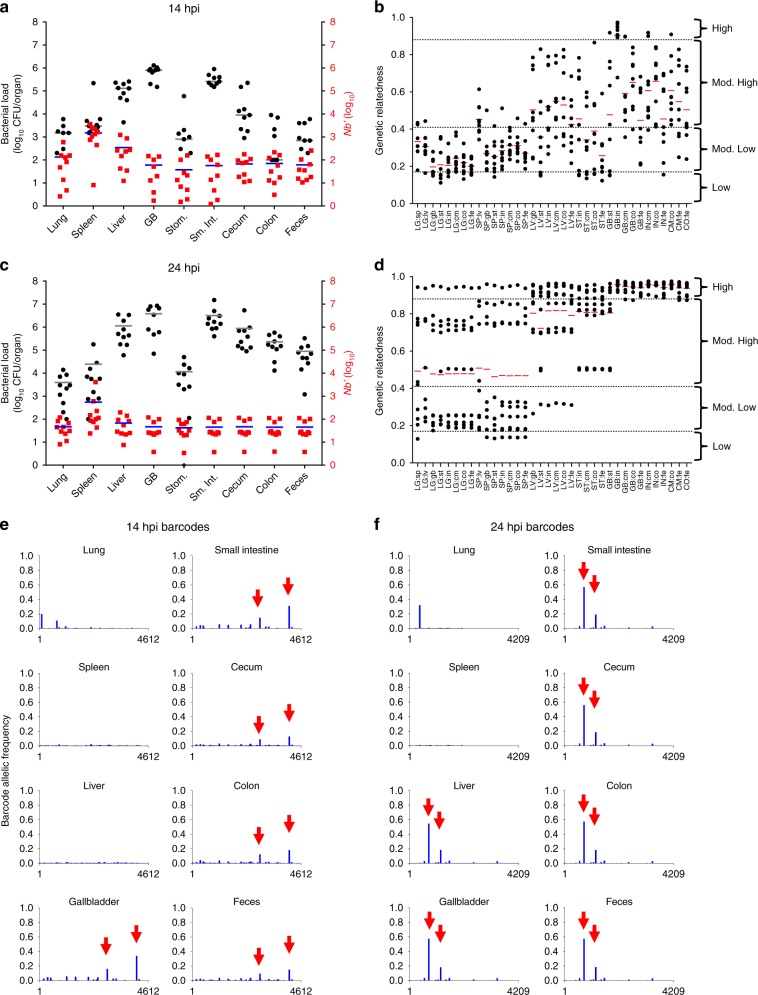
Diversity and relatedness *P. aeruginosa* populations following bloodstream infection. BALB/c mice were intravenously injected with ~2 × 10^6^ CFU of PABL012_pool_. **a** Bacterial loads (total CFU per organ, black circles) and *N*_*b*_′ sizes (red squares) in different tissues were determined at 14 (**a**) and 24 hpi (**c**). CFU values are expressed per organ for the lung, spleen, liver, gallbladder (GB), stomach (Stom.), small intestine (Sm. Int.), cecum, colon, and feces. Each black circle and red square represent an organ from one mouse (14 hpi *n* = 9, 24 hpi *n* = 10). Geometric means (horizontal lines) are shown. Genetic relatedness (GR) of PA populations at 14 (**b**) and 24 hpi (**d**). Each circle represents one organ in pairwise comparison with another organ from the same mouse. Geometric means (red, horizontal bars) are shown. Dashed lines denote genetic relatedness categories, which are defined as High (≥0.88), Moderately High (Mod. High, <0.88, ≥0.41), Moderately Low (Mod. Low, <0.41, ≥0.17), and Low (<0.17). LG (lung), SP (spleen), LV (liver), ST (stomach), GB (gallbladder), IN (small intestine), CM (cecum), CO (colon), FE (feces). The relative allelic frequencies of all unique barcodes in every organ of a representative mouse is shown at 14 (**e** *n* = 4612 total barcodes) and 24 hpi (**f** *n* = 4208 total barcodes). Red arrows denote the top 2 dominant barcodes in the LV, GB, IN, CM, CO, and FE.

We applied the optimized STAMP approach to mice with PA bacteremia. Mice (*n* = 10) were injected with PABL012_pool_; per-organ total CFU counts recovered were consistent with those observed in prior experiments (Fig. 2a, c compared to Fig. 1k, l). At 14 and 24 hpi, the mean founding population sizes (*N*_*b*_ʹ) were quite low (between 35–70 at 14 hpi and 40–60 at 24 hpi) in the majority of the organs. In contrast, the spleen had a larger mean *N*_*b*_ʹ of 1509 at 14 hpi (Fig. 2a) and 523 at 24 hpi (Fig. 2c), indicative of a more diverse founding population that likely reflects the role of the spleen in filtering bacteria from the bloodstream. The low *N*_*b*_ʹ values of the GB and intestines suggest that PA experiences a severe bottleneck in moving from the bloodstream to these organs. This low diversity (*N*_*b*_ʹ) coupled with a high bacterial density (CFU), suggests that the few PA cells that traverse this narrow bottleneck are able to replicate to high levels upon arrival in the GB.

Interestingly, the founding population sizes for PA isolated from the liver, GB, intestines and feces were all quite similar, suggesting that these bacterial populations may be related to one another. We hypothesized that the liver may be imposing the tight bottleneck observed in our *N*_*b*_ʹ calculations. Because of the anatomic link between the liver, GB, and intestines, PA bacteria may migrate from the liver to the GB, where they undergo unimpeded replication and subsequent escape from the host via the intestinal tract. To address this hypothesis, specific barcode frequencies (allelic frequencies) of the bacterial populations from pairs of organs taken from individual mice were compared to determine their genetic relatedness (GR) according to the method of Cavalli and Sforza^12^ (Fig. 2b, d). The PA populations within the GB, small intestine, cecum, colon, and feces of each mouse increased in relatedness from 14 hpi (Fig. 2b) to virtually identical populations by 24 hpi (GR values approaching 1, Fig. 2d). In silico simulations revealed that the high levels of GR observed in our animal experiments were not secondary to chance or influenced by the skewed distribution of barcodes in PABL012_pool_ (Supplemental Figs. 5b, 6b), but suggested that the populations were linked to each other (Supplementary Fig. 7, see Methods section for details). Moreover, at both 14 and 24 hpi, these populations were dominated by the same relatively few sequence barcodes (Fig. 2e, f, Supplementary Figs. 5a and 6a), indicating that a relatively small number of bacteria founded this population and subsequently replicated to high levels. These dominant sequence tags were consistently the dominant sequence tags from the liver (Supplementary Figs. 5a and 6a), suggesting that the population in the GB arose from a subpopulation of cells that migrated from the liver. Taken together, these data suggest that during bacteremia a small subpopulation of PA replicated in the liver and seeded the GB. Once in the GB this population then expanded dramatically, disseminated to the intestines and was excreted in the feces. The lack of high levels of GR between the populations in the lung, spleen and liver suggests that these organs were seeded independently by unique founding populations and that the dynamics of population control within each organ were different (Fig. 2b, d).

### PA is shed in the feces of bacteremic mice for up to 10 days

**Fig. 3:**
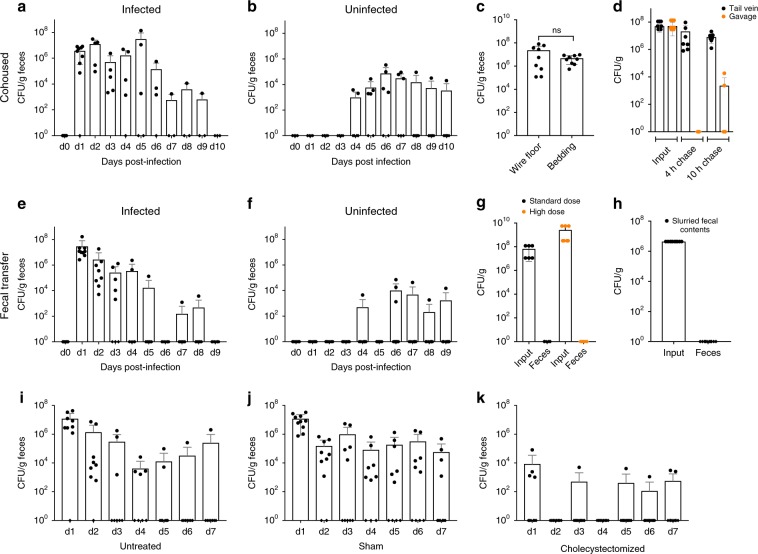
The gallbladder promotes prolonged intestinal shedding of *P. aeruginosa* following bacteremia. BALB/c mice were injected with ~8 × 10^5^ CFU of PABL012_lux_ (**a**) and co-housed with 2 uninfected cage-mates (**b**) for 10 days (*n* = 3 x 3 replicates infected, *n* = 2 x 3 replicates uninfected). Fecal pellets were collected from individual mice daily. **c** Mice intravenously infected with ~2 × 10^6^ CFU of PABL012_lux_ were housed on normal bedding or raised wire racks (*n* = 3 × 3 replicates), and PA was measured in the feces at 24 hpi. (ns) not significant by Mann–Whitney test. **d** Mice were infected intravenously with ~2 × 10^6^ CFU of PABL012_lux_ (black circles), followed 4 h (*n* = 7 total, 2 replicates) or 10 h (*n* = 9 total, 2 replicates) later with oral gavage of ~2 × 10^6^ CFU of PABL012_GM_ (orange circles) and PA was measured in the feces at 24 hpi. BALB/c mice (*n* = 4 x 2 replicates) were injected with ~8 x 10^5^ CFU of PABL012_lux_  and all excreted fecal pellets from infected mice (**e**, *n* = 8) were transferred daily to the cages of uninfected mice (**f**, *n* = 9). Fecal pellets were collected from individual mice daily. **g** Mice were orally administered standard (~2 × 10^6^ CFU, *n* = 3 × 2 replicates, black circles) or high (~3 × 10^8^ CFU, *n* = 4 × 2 replicates, orange circles) doses of PABL012_lux_ by gavage, and PA was measured in the feces at 24 hpi. **h** Fecal pellets from previously infected mice (*n* = 25) were slurried and orally administered (~2 × 10^5^ CFU) to uninfected mice by gavage. PA was measured in the feces at 24 hpi. For input 1 g equals 1 mL of wet weight. **i**–**k** Mice (*n* = 5 x 2 replicates) following sham surgery (**j**) and mice following cholecystectomy (**k**) and mice that had not undergone surgery (**i**), were challenged intravenously with ~2 × 10^6^ CFU of PABL012_lux_ and fecal pellets collected daily. On day 1, *p* values were determined for wild type vs. sham (*p* = 0.9, ns), wild type vs. cholecystectomized (**p = 0.004), and sham vs. cholecystectomized (***p = 0.0005) mice via a Kruskal–Wallis analysis to correct for multiple comparisons. For scatter plots, each circle (or diamond) represents data from one mouse, and data are presented as PA shed in CFU per gram feces [CFU/g]. When no bacterial CFU were recovered, data are represented as diamonds on the *x*-axis. Geometric means (boxes), and SD (whiskers) are shown.

We next examined the duration of PA excretion following bloodstream infection. Mice were infected with a sublethal infectious dose (~8 × 10^5^ CFU) of PABL012_lux_. By 2 days post infection, the feces of 100% of infected mice contained between 10^4^ and 10^8^ CFU per gram of feces of PABL012_lux_, and excretion was sustained over 5 days (Fig. 3a). Beginning at day 6 and continuing through the remainder of the experiment, the number of infected mice shedding this concentration of PABL012_lux_ dramatically declined until all infected mice cleared PA from their stools by day 10 (Fig. 3a). The levels of PA in the feces of infected mice were not influenced by the coprophagic behavior of mice, as infected mice housed on raised wire floors (whereby fecal pellets are inaccessible for ingestion) showed no difference in the fecal bacterial load at 24 hpi compared to infected mice housed on normal bedding (Fig. 3c). To further verify that the observed fecal shedding of bacteremic mice was not a consequence of coprophagia, we tested whether orogastrically delivered PA contributed to the PA load in the feces of bacteremic mice. A PA strain marked with a chromosomal gentamicin resistance cassette (PABL012_GM_) was administered by oral gavage to mice systemically infected either 4 or 10 h prior with PABL012_lux_ (Fig. 3d). The orally administered strain could only be detected in the feces of two mice when administered 10 h after initial injection of PABL012_lux_ (Fig. 3d). Furthermore, this recovered population of PABL012_GM_ was 1000 times smaller than the fecal population of systemically delivered bacteria (PABL012_lux_) and had a minimal contribution to the overall bacterial counts in the feces. Together, these experiments indicate that bacteremic mice shed substantial amounts of PA into their environment regardless of mouse coprophagic behaviors.

### Fecal PA is transmitted to uninfected mice

We next examined the consequences of such high levels of PA fecal excretion into the local environment on transmission. Mice infected intravenously with PABL012_lux_ (~8 × 10^5^ CFU) were co-housed with uninfected mice, and both sets of mice were monitored daily for outward signs of illness and fecal excretion of PA. Daily cage and water changes were performed to minimize any potential environmental PA reservoirs. Although no uninfected mice displayed any outward signs of PA infection, approximately 60% of the uninfected mice began to excrete PABL012_lux_ (10^3^–10^4^ CFU per gram feces) by day 4, with peak fecal excretion by uninfected mice on day 6 (Fig. 3b). This was followed by a steady decline in bacterial counts in the feces over the remaining 4 days. Moreover, one uninfected mouse continued to excrete PABL012_lux_ at the end of the experiment despite clearance from the originally infected animals, suggesting that this mouse maintained prolonged carriage of PA in the GI tract. These experiments revealed that PA excreted by systemically infected mice may facilitate transmission to uninfected co-housed mice that manifests as detectable numbers of PA in the feces.

We hypothesized that PABL012_lux_ detected in the feces of uninfected mice resulted from ingestion of contaminated fecal pellets shed by infected mice. This observation was somewhat surprising, given our previous results demonstrating the negligible impact of coprophagia on PA in the feces (Fig. 3c, d). Likewise, previous work in mouse models demonstrated that PA cannot access the GI tract through orogastric routes unless extremely high doses of bacteria (>10^7^ CFU per mL drinking water) were administered to antibiotic treated animals depleted of GI microbiota^13–15^. To further explore this phenomenon, we orogastrically delivered large numbers of media-grown PABL012_lux_ to uninfected mice and observed no detectable PA in the feces of these mice at 24-hours post-gavage (Fig. 3g). To test whether the material and microbes present in feces were necessary to facilitate PA carriage, we homogenized PABL012_lux_-contaminated fecal pellets into a slurry and orally inoculated the slurry (2 × 10^5^ CFU, maximum obtainable dose from pellets of ≈25 shedding mice) into mice. No bacteria could be detected in the feces of these mice at 24-hours post-gavage (Fig. 3h), indicating that the presence of contaminated fecal material is not sufficient to allow orally administered PA to result in carriage. We, therefore, hypothesized that the nature of the intact fecal pellet in some way facilitates this process. For example, the deeper portions of the pellet may enhance the survival of anaerobic bacteria that promote PA carriage in the GI tract or the pellet architecture might enable PA survival during passage through the acidic environment of the stomach. To address this, we collected all fecal pellets excreted daily from mice infected intravenously with PABL012_lux_ and transferred only the contaminated fecal pellets to the cages of uninfected mice simultaneously with daily cage and water changes. Although the numbers of fecal PA ingested by the uninfected mice could not be determined, it was estimated to be significantly less than the orogastric doses used in the preceding experiments. One uninfected mouse began to excrete PABL012_lux_ by day 4 and additional mice by day 6 (10^3^–10^4^ CFU per gram feces, Fig. 3e, f). These findings confirm that exposure to PA contained within fecal pellets is sufficient to facilitate transmission to uninfected animals. In summary, fecal excretion of PA by bacteremic mice is sufficient to allow transmission, which is manifested by detectable numbers of PA in the feces of naïve mice.

### The GB is crucial for fecal excretion of PA

Given the results of our STAMP analysis indicating that the population of PA excreted in the feces was preceded by dramatic expansion in the GB, we wondered what impact removal of the GB would have on excretion. Untreated (wild type), sham (peritoneum opened and repaired), and cholecystectomized mice (*n* = 5x 2 replicates) were infected with the standard dose (2 × 10^6^ CFU) of PABL012_lux_. Daily cage and water changes were performed to minimize any environmental contamination. On day 1, 90% of the untreated and 100% of the sham mice excreted high levels of PABL012_lux_ (between 10^6^ and 10^8^ CFU per gram feces), whereas only 40% of the cholecystectomized mice excreted significantly less PABL012_lux_ (between 10^3^ and 10^5^ CFU per gram feces). The proportion of all excreting mice decreased through day 7; however, of the excreting mice, the untreated and sham mice continued to excrete higher levels of PA than cholecystectomized mice (Fig. 3i–k). These results indicate that the bacterial expansion of PA in the GB is crucial for high levels of bacterial excretion into the environment.

### Type III secretion dramatically impacts PA dissemination

**Fig. 4:**
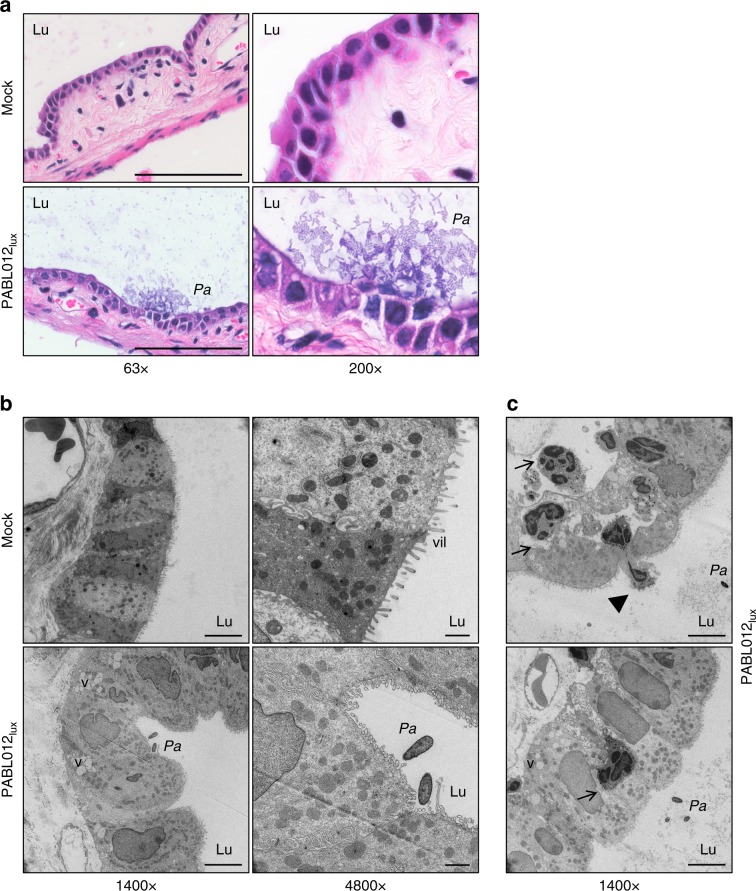
*P. aeruginosa* infection damages the gallbladder epithelium. BALB/c mice intravenously infected with PABL012_lux_ (~2 × 10^6^ CFU) were euthanized at 24 hpi to harvest gallbladders for histological preparation. **a** Hematoxylin & eosin (H&E) staining of mock (PBS) or PABL012_lux_-infected mouse gallbladder sections at ×63 and ×100 (scale bar = 100 µM). **b**, **c** TEM of gallbladder sections from mock (PBS) or PABL012_lux_-infected mice (scale bars = 5 µM (×1400), 1 µM (×4800)). Black arrows indicate neutrophils, the black arrowhead shows a transmigrating neutrophil extravasating into the gallbladder lumen. Lu (lumen), V (vacuoles), vil (villi), *Pa* (*P. aeruginosa)*.

We sought to determine whether the high numbers of PA observed in the GB resulted in damage to the epithelium of this organ and whether known PA virulence factors played a role in this process. To do so, we examined GBs of infected animals for histologic and ultrastructural evidence of injury. GBs from mock-infected animals, having no visible bacteria in the lumen, had a typical mucosal layer consisting of columnar epithelial cells with basal nuclei, extended microvilli, and an intact lamina propria (Fig. 4a, b). In contrast, the mucosa of GBs from infected mice displayed a general distortion of the cellular architecture characterized by large nuclei, widened tight junctions, increased vacuoles, and disrupted lamina propria (Fig. 4a, b). Disrupted microvillus architecture, characterized by blunted, disordered, or absent microvilli, was observed at the epithelial surface, especially in areas of close association with PA (Fig. 4b). In mice infected with PABL012_lux_, we also noted intermittent focal bacterial aggregates at the epithelial surface overlying damaged columnar epithelial cells (Fig. 4a, *Pa*). Neutrophils were observed at both the basolateral surface (arrows) of the epithelial layer and extravasating through it toward visible lumenal bacteria (arrowhead) (Fig. 4c).

**Fig. 5:**
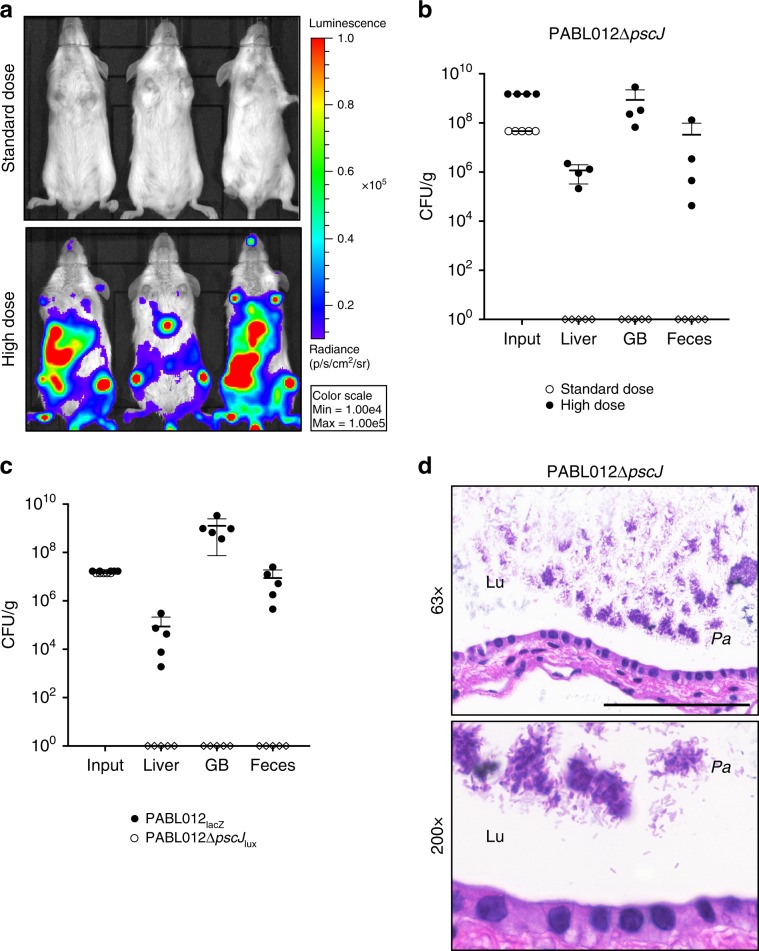
The *P. aeruginosa* type III secretion system (T3SS) contributes to gastrointestinal shedding. **a** BALB/c mice (*n* = 3, representative replicate) were infected intravenously with a standard (~2 × 10^6^ CFU, open circles) or a high dose (~8 × 10^7^ CFU, filled circles) of a PABL012∆*pscJ*_lux_ T3SS mutant and imaged at 24 hpi using IVIS. The scale (red = high, blue = low) for all images represents radiance (photons per sec per cm^2^ per steradian) with minimum and maximum values normalized to 1 × 10^4^ and 1 × 10^5^, respectively. **b** Mice (standard dose *n* = 5, open circles; high dose *n* = 4, black circles) were euthanized, bacteria enumerated from the liver, gallbladder (GB), and feces, and CFU per gram organ reported at 24 hpi. **c** BALB/c mice (*n* = 5) were co-infected intravenously with a 1:1 mix totaling ~2 × 10^6^ CFU of PABL012_lacZ_ (*n* = 5, filled black circles) and PABL012∆*pscJ*_lux_ (*n* = 5, open symbols). Liver, GB, and feces were harvested at 24 hpi for enumeration of viable bacteria. For input 1 g equals 1 mL of wet weight. For scatter plots, each circle or diamond represents data from one mouse. Where no CFU were recovered, data are represented as diamonds on the *x*-axis. Geometric means (horizontal lines) and SD (whiskers) are shown. **d** H&E stained sections of gallbladders from mice infected with the high dose of PABL012∆*pscJ*_lux_. Lu (lumen), *Pa* (*P. aeruginosa)*. Scale bar represents 100 µM (×63).

The type III secretion system (T3SS) is a major driver of virulence in PA and is known to damage epithelial surfaces^16,17^ through direct injection of cytotoxic effector proteins. To assess the role of the T3SS in expansion within the GB and intestines, mice were infected with a T3SS mutant (PABL012∆*pscJ*_lux_) and monitored for disease progression. The T3SS mutant was absent from the liver, GB, and feces when infected at the standard wild-type dose (Fig. 5a, b). In addition, the PABL012∆*pscJ*_lux_ mutant phenotype could not be rescued by coinfection with wild-type PABL012_lux_ (Fig. 5c), suggesting that any in vivo bottlenecks act directly on the bacteria. The attenuation of the T3SS mutant could be overcome by increasing the infectious dose 100-fold (Fig. 5a, b). However, in contrast to the mucosal damage observed with wild-type infection, no damage to the GB epithelium was observed at this higher dose (Fig. 5d). Thus, an intact T3SS is important for PA dissemination to the GB and subsequent fecal shedding, and results in damage to the GB epithelium.

### PA traffics to the GB and is excreted following pneumonia

While PA is a frequent cause of bloodstream infections, it is also an important cause of ventilator-associated pneumonia^18^, and previous studies have demonstrated that PA is capable of escaping into the bloodstream during pneumonia^19^. Given our observations following direct inoculation of PA into the bloodstream, we hypothesized that, following pneumonia, PA would disseminate to and expand within the GB. To determine whether GB expansion and fecal excretion were observed following PA pneumonia, 6- to 8-week-old BALB/c mice were infected by intranasal inoculation with PABL012_lux_ (standard dose, 2 × 10^6^ CFU) and monitored for 24 hpi. Infected mice displayed a largely disseminated pattern of infection (Fig. 1m) and contained high numbers of bacteria in the liver, GB, and feces (Fig. 1n). These data suggest that following pneumonia, presumably through direct access to the bloodstream, PA can also expand within the liver and GB, and be ultimately excreted in the feces. Thus, both primary and secondary bloodstream infections lead to the excretion of high levels of PA into the environment.

## Discussion

**Fig. 6:**
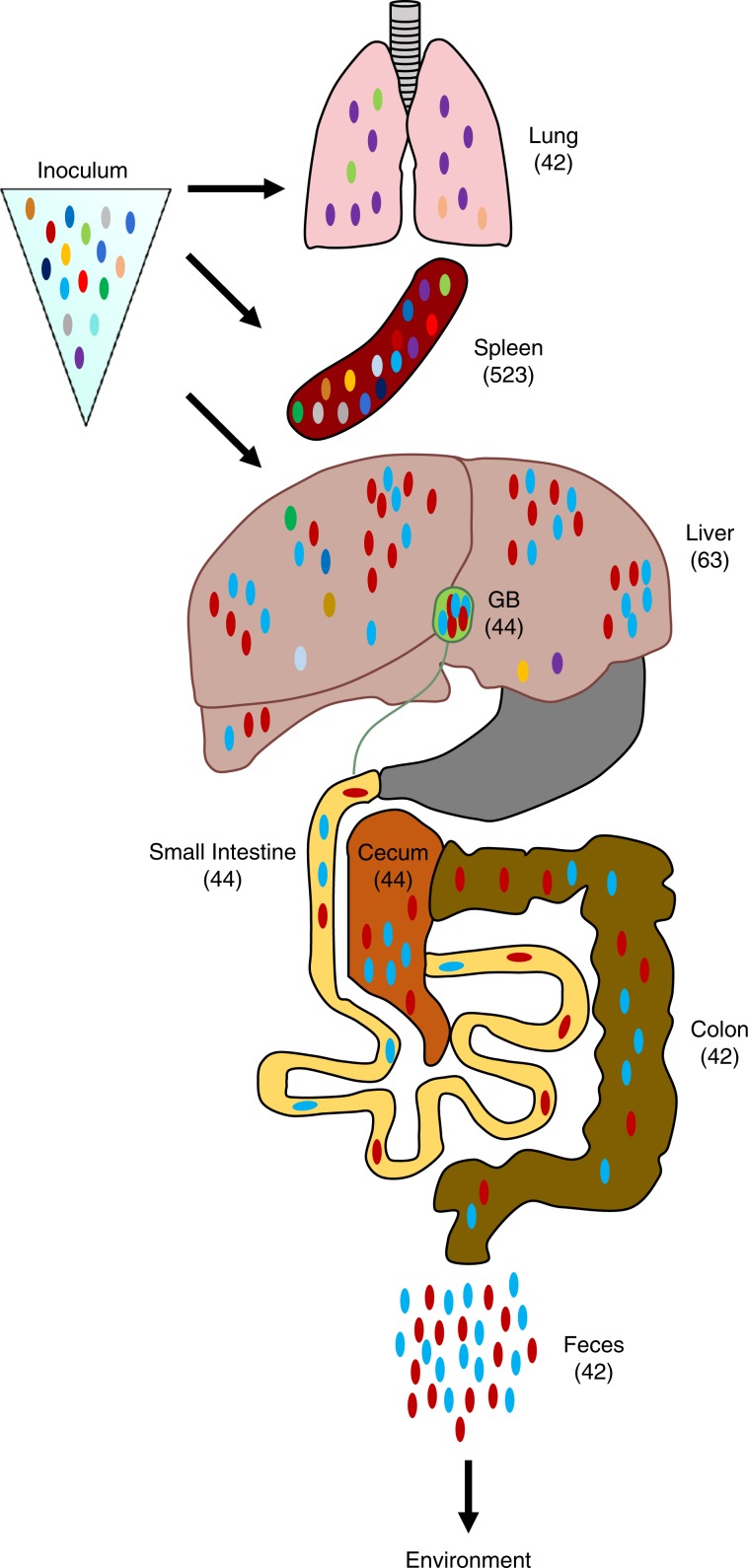
Model of *P. aeruginosa* population dynamics following bloodstream infection. PA populations with unique complexity arise in the lungs, liver, and spleen of intravenously infected animals. The bottleneck sizes at 24 hpi within the liver and lung are similar (mean *N*_b_′ values for all mice are shown in parentheses); however, their founding populations are distinct. Very few organisms establish infection in the gallbladder (GB), but the founders ultimately expand to very high numbers. Bacteria released from the GB through the bile into the small intestine become the principal source of PA excreted in the feces.

PA infections are a threat to global health due to their severity and the increasing frequency of MDR strains. For example, PA bloodstream infections are associated with mortality rates between 25 and 50%^2,20,21^. By applying in vivo imaging of bioluminescent bacteria and STAMP analysis to a mouse model of bacteremia, we have identified an unexpected pathway through which PA experiences a narrow bottleneck as it traffics from the bloodstream to the GB and ultimately the intestines. The GB serves as protected expansion niche for PA that facilitates excretion into the environment (Fig. 6). In epidemiologic studies in humans and mice, the GB has been shown to be an important replication niche for a variety of enteric pathogens including *Listeria monocytogenes (Lm)*, *Escherichia coli*, and *Salmonella enterica* serovar Typhi^7,22,23^, and replication in the GB has been shown to fuel transmission through GI excretion. STAMP was previously applied to *Lm* bloodstream infection revealing that *Lm* experiences a similarly narrow bottleneck in the GB and intestines^9^ and identifying the GB as the main driver of fecal excretion. Here, we show that a similar phenomenon occurs with the non-enteric and healthcare-associated pathogen PA. PA migrates through a narrow bottleneck to reach the GB, and removal of this organ dramatically reduces the levels of PA excreted into the environment. Thus, GB expansion of a small population of bacteria following bloodstream infection is a strategy more universally employed by bacteria than previously appreciated.

Our findings suggest that PA is remarkably well equipped for replication in the GB. Bile and bile salts have traditionally been viewed as toxic to bacteria^24^; however, both laboratory-adapted and clinically derived PA strains expanded in the GBs of mice, and select strains grew ex vivo in bile as well as in enriched medium. The mechanisms of bile resistance in PA are unknown; however, a role for multidrug efflux pumps such as MexAB-OprM has been suggested^25,26^. Thus, bile exposure may promote antibiotic resistance by inducing expression of efflux pumps, and transit through the GB may enhance excretion of drug-resistant PA. Hardy and colleagues suggested that *Lm* uses the lumen of the GB to escape immune surveillance^22^, and it is possible that the immune privileged nature of this organ provides an ideal environment for PA replication as well. PA plays an active role in promoting trafficking to and expansion in the GB as these do not occur in the absence of a functional T3SS. These findings suggest that, in addition to enhancing virulence, the PA T3SS functions to promote GI carriage and transmission.

As an opportunistic pathogen, PA is occasionally carried in the healthy human GI tract and can systemically infect humans when barrier defenses are compromised. In patients, such systemic infections are viewed as evolutionary and ecological “dead-ends” in that they are disadvantageous for pathogen survival. Our work challenges this paradigm in the context of mouse infections. Once in the bloodstream, PA enacts a virulence strategy whereby it exploits the GB as a protected replication niche and facilitates its own exit back into the environment via high levels of intestinal excretion. Thus, PA bloodstream infections are not an ecological “dead-end” but rather lead to enhanced GI carriage and fecal excretion which serves as a PA survival and transmission strategy (Fig. 6).

Our results were obtained using a murine model, but it is intriguing to speculate whether they apply to humans. A number of observations indicate that PA can access the GBs of patients. PA is one of the five pathogens most commonly isolated from the bile of individuals with cholecystitis^27^ and has been associated with secondary sclerosing cholangitis, a dreaded complication of critical illness that involves the biliary system of the liver^28^. Likewise, PA is a leading cause of acalculous cholecystitis (inflammation of the GB in the absence of gallstones), a common complication of serious medical illness^29^. In these contexts, it has been suggested that PA gains access to the biliary tree by ascending from the duodenum via the common bile duct. However, it is conceivable that some of these infections are secondary to occult or intermittent PA bacteremia and arrive in the GB via the liver. Likewise, the GI tract is recognized as an important reservoir for PA in hospitalized patients^30–32^, and it is appreciated that PA GI carriage is a precursor to both invasive infections^33,34^ and environmental contamination. Could this environmental contamination lead to transmission of PA between patients? Transmission of PA via the hands of health care workers^35–38^ or indirectly through contamination of environmental objects such as bedrails, sinks, drains, and endoscopes^39,40^ has been repeatedly documented and postulated to explain the geographic spread of global epidemic strains, such as sequence type (ST) 111, ST175, and ST235^41,42^. With regard to transmission via fecal excretion, an obvious difference between mice and people is that the latter are not coprophagic. Yet, fecal–oral spread of other bacterial pathogens such as *Clostridioides difficile* and vancomycin-resistant enterococci, is clearly common among patients in intensive care units and the cause of considerable infection control efforts. Ample opportunities may therefore exist for PA-contaminated fecal material to also spread from patient-to-patient. However, whether such transferred material contains sufficient numbers of PA and whether it is in a form that facilitates transmission (Fig. 3) is unclear. Additional studies are necessary to examine the role of the GB and intestinal tract in transmission of PA among patients.

Ultimately, this work identified bacterial expansion in the mouse GB by the environmentally ubiquitous, non-enteric, opportunistic pathogen PA and suggests that the strategy of hijacking the GB as a protected site for bacterial expansion to facilitate high levels of fecal excretion may be more widely utilized by bacterial species than previously thought. In this model system, PA bacteremia led to GB expansion and fecal excretion, challenging the paradigm that bloodstream infections are “dead-ends.” Removal of the GB dramatically reduced PA excretion. Our work highlights an urgent need to better understand the role of the GB in transmission of this highly antibiotic-resistant bacterial pathogen.

## Methods

### Bacterial strains and growth conditions

*P. aeruginosa* PABL002, PABL012, PABL016, PABL017, PABL028, PABL030, PABL041, PABL046, PABL049, PABL095, and PABL107 are clinical isolates cultured from the bloodstream of patients at Northwestern Memorial Hospital between September 1, 1999 and June 9, 2002^20^. PA14 and PAO1 are commonly used laboratory strains^43,44^. PAC6 and S10 are clinical isolates from the bloodstream of patients in Taiwan with Shanghai Fever^15^. Relevant characteristics of these strains are listed in Supplementary Table 1, and a parsimony tree based on single-nucleotide polymorphism (SNP) loci present in 95% of the genomes generated using kSNP v3.0.21^45^ and visualized in iTOL^46^ is presented (Supplementary Fig. 3).

*Escherichia coli* strain TOP-10 (Invitrogen) was used for cloning and *E. coli* strains S17.1 λpir^47^ and SM10 λpir (gift of John Mekalanos, Harvard Medical School) were used to introduce plasmids into *P. aeruginosa*. Bacterial strains were streaked from frozen cultures onto either Vogel–Bonner minimal (VBM) agar^48^ or LB agar and, unless otherwise stated, grown at 37 °C in either LB or MINS media^49^.

Antibiotics were used at the following concentrations: irgasan 5 µg per mL (irg), gentamicin 100 μg per mL (gm), carbenicillin 250 μg per mL, and tetracycline 75 μg per mL (tet) for PA and gentamicin 15 μg per mL, carbenicillin 50 μg per mL, and tetracycline 10 μg per mL for *E. coli*. For blue–white discrimination, 5-bromo-4-Chloro-3-Indolyl-d-Galactopyranoside (Xgal) was added to plates at a concentration of 80 μg per mL. Further details on the strains and plasmids used in this study can be found in Supplementary Tables 1–3.

### Generation of labeled *P. aeruginosa* strains

The plasmid pminiCTXnpt2lux^50,51^, was transformed into *E. coli* strain S17.1 λpir. Following conjugation, the luciferase cassette was introduced into the chromosomal *attB* site of the *P. aeruginosa* strains PAO1, PA14, PABL012, and PABL012∆*pscJ*, PABL002, PABL016, PABL017, PABL028, PABL030, PABL041, PABL046, PABL049, PABL095, PABL107, PAC6, and S10 via integrase-mediated recombination using previously defined approaches^52^ to generate luciferase-labeled strains (e.g., PABL012_lux_). Bioluminescent bacteria were confirmed by imaging plates on the IVIS Lumina LTE^®^ in vivo Imaging System.

The gentamicin resistance cassette, including its native promoter, was amplified from pEX18.Gm^53^ with the primers Gm-pCTX_F-BamHI-2 and Gm-pCTX_R-EcoRI (Supplementary Table 4). It was then ligated into the multiple cloning site of pminiCTX-1^52^ to generate the plasmid pminiCTX-1_GM_. As above, the resulting plasmid was transformed into *E. coli* S17.1 λpir and, following conjugation, was introduced into PABL012 to generate the strain, PABL012_GM_.

The pminiCTX-1_lacZ_ plasmid (gift from Stephen Lory, Harvard Medical School) was transformed into *E. coli* strain SM10 λpir. Following conjugation, the *lacZ* gene was introduced into the *attB* site of strain PABL012 via integrase-mediated recombination using previously described approaches to generate the strain, PABL012_lacZ_.

### Generation of type-3-secretion-deficient *P. aeruginosa*

Upstream and downstream fragments surrounding the *pscJ* gene were amplified from PAO1 genomic DNA using the following primers: pscJ-5-1-HindIII, pscJ-5-2, pscJ-3-1, and pscJ-3-2 HindIII, where pscJ-5-2 and pscJ-3-1 contain a 24 bp-overlapping linker sequence (*TTCAGCATGCTTGCGGCTCGAGTT*) to generate an in-frame deletion of the *pscJ* gene (Supplementary Table 4). The integration proficient vector, pEXG2^54^ was cut with HindIII and the three fragments were ligated using the New England Biolabs Gibson Assembly^®^ Cloning Kit. The resulting vector, pEXG2∆*pscJ*, was verified by sequencing at the NuSeq facility at Northwestern University and transformed into *E. coli* SM10 λpir. Following conjugation and allelic exchange with PABL012_lux_, whole-genome sequencing was performed on strain PABL012∆*pscJ*_lux_ to confirm the mutation.

### Mouse model of tail vein injection

Overnight cultures of PA were grown in 5 mL of MINS medium, diluted into fresh medium the next day, and regrown to exponential phase prior to infections. The tail veins of female 6- to 8-week-old BALB/c mice restrained using a TV-150 Tailveiner^®^ (Braintree Scientific) were dilated with a heat lamp. A defined number of PA bacteria in 50 µL of phosphate-buffered saline (PBS) was injected into the tail veins of mice using a 27-gauge needle. Inoculums were confirmed by plating serial dilutions on LB agar. To monitor bacterial excretion, fecal pellets were harvested individually from each mouse, weighed, and resuspended in PBS. At specified times postinfection, the mice were anesthetized and sacrificed by cervical dislocation. For dissemination experiments, organs were excised, weighed, and homogenized in PBS using the Benchmark^®^ Bead Blaster 24. Viable bacteria were enumerated by plating serial dilutions of either fecal pellets or organs on LB agar containing 5 μg per mL of irgasan to select for PA. Luminescent bacteria were confirmed by imaging plates on the IVIS Lumina LTE^®^ in vivo Imaging System. Recovered colonies of bacteria were counted to determine total CFU or CFU per gram organ.

Animals were purchased from Harlan Laboratories, Inc. (BALB/c, Indianapolis, IN) and Jackson Laboratory (C57/BL6, Bar Harbor, ME) and housed in the containment ward of the Center for Comparative Medicine at Northwestern University. All experiments were approved by the Northwestern University Institutional Animal Care and Use Committee in compliance with all relevant ethical regulations for animal testing and research.

### Mouse model of oral gavage

Briefly, overnight cultures of PA were grown in 5 mL of MINS medium, diluted into fresh medium the next day, and regrown to exponential phase prior to infections. Mice were restrained, and a 20-gauge blunt-end straight feeding needle (Pet Surgical, Sherman Oaks, CA) was used to gavage 50 µL of the appropriate dose of PA directly into the stomachs of 6- to 8-week-old BALB/c mice. Again, inoculums were confirmed by plating serial dilutions on LB agar. Organ harvesting and bacterial plating for CFU enumeration was performed as described above.

### Mouse model of acute pneumonia

The mouse model of acute pneumonia described by Comolli et al.^55^ was used for all animal experiments. Briefly, 6- to 8-week-old female BALB/c mice were anesthetized by intraperitoneal injection of a mixture of ketamine (75 mg per kg) and xylazine (5 mg per kg). Mice were intranasally inoculated with specified doses of bacteria in 50 μL of PBS. Inoculums were confirmed by plating serial dilutions on LB agar. Organ harvesting and bacterial plating for CFU enumeration was performed as described above.

### Mouse cholecystectomy and sham surgery

Briefly, 4- to 6-week old BALB/c mice were transported to the Northwestern Microsurgery Core at Northwestern University. There, mice were anaesthetized with a single dose of ketamine (100 mg per kg) and xylazine (20 mg per kg) and administered a subcutaneous one-time dose of 0.05 mg per kg buprenorphine prior to incision. Nair was used to remove abdominal hair, and the mouse was positioned on its dorsal surface under an operating microscope. A 1-in. skin incision was made in the abdominal skin followed by a similar incision in the peritoneum. The peritoneum was reflected and the sternum retracted. Bowels were covered in sterile gauze infused with warm saline. The ligament attaching the murine GB to the base of the diaphragm was severed with electrocautery. The GB was then tied off at the base with an absorbable 7-0 silk suture, excised in total with electrocautery. The abdominal cavity was irrigated with warm saline and the peritoneum closed with a 5-0 absorbable suture. The abdominal skin was closed with a 5-0 nylon, non-absorbable suture using a running stitch. Mice were given an immediate postoperative dose of subcutaneous meloxicam (1 mg per kg) and recovered at 32 °C for 24 h. At 24-h post-operation, mice were given an additional one-time subcutaneous dose of 1 mg per kg of meloxicam. For “sham” mice, the operative protocol was identical to above with the exception that GBs were not removed. Mice recovered for one week postoperatively prior to challenge with PA in the containment ward of the Center for Comparative Medicine at Northwestern University. During this phase, mice were monitored daily for signs of distress and infection.

### In vivo localization of bacteria using the IVIS imaging system

Representative 6- to 8-week-old female BALB/c mice were challenged either intravascularly or intranasally with various bioluminescent PA strains. At specific times postinfection, mice were anesthetized with vaporized isoflurane using the XGI-8 Gas Anesthesia System, transferred to the stage of the IVIS Lumina LTE^®^ in vivo Imaging System, and both photographic and bioluminescent images were captured. All images were captured with an exposure time of 2 min and medium binning. Bioluminescence is represented as a heat map (red =  high, blue = low) normalized to a range of 1 × 10^4^ to 1 × 10^5^ radiance (photons per second per cm^2^ per steradian). Images were processed with Living Image^®^ Software version v4.0 by Caliper LifeSciences.

### Construction of a library of barcoded *P. aeruginosa*

The STAMP protocol used here was similar to that described by Abel et al. and Zhang et al.^9,11^. Supplementary Table 4 contains the sequences of all primers used in this protocol. The plasmid pminiCTX_STAMP_ used for generating the tagged *P. aeruginosa* library was constructed as follows. The gentamicin resistance cassette (~1032 bp) from pEX18.Gm^53^ was amplified with primers P110 (which contains the barcodes) and P80^11^ (Supplementary Table 4) and inserted into the EcoRI site of the integration proficient plasmid pminiCTX-1^52^. The resulting plasmid, pminiCTX_STAMP_, was transformed into *E. coli* strain TOP-10 and selected with gentamicin. Plasmid DNA was harvested and transformed into *E. coli* SM10 λpir. Following conjugation, pminiCTX_STAMP_ was introduced into the *attB* site of the *P. aeruginosa* strain PABL012 via integrase-mediated recombination to generate PABL012_pool_, and transconjugants were selected with tetracycline. After 10 of 10 tested colonies were found to have a unique, correct insertion of pminiCTX_STAMP_, the remaining colonies were scraped off plates and pooled. After addition of 25% glycerol, the pooled library of PABL012, PABL012_pool_ was divided into aliquots and stored at −80 °C. The plasmid was stably integrated for at least 20 h without selection (Supplementary Fig. 5b).

### Calibration curve for the STAMP study

As described by Abel et al.^11^, the diversity of barcodes present in a subculture of tagged bacteria can be used to calculate the size of the founding population of that subculture. We compared the mathematically determined founding population sizes to those induced experimentally (*N*_*b*_). Briefly, we diluted three frozen aliquots of the PABL012_pool_ library (A–C) (50 μL) 1:100 in MINS and grew with shaking overnight at 37 °C. In the morning, cultures were diluted into fresh MINS medium and grown at 37 °C for 3 h. Cells were harvested by centrifugation (13,000×*g*, room temperature, 2 min) and resuspended in PBS. A small portion of each serial dilution of each replicate was used to enumerate total CFU, and the remainder was plated at 37 °C overnight. Following growth, all bacterial colonies were scraped off using Falcon cell scrapers into 5 mL of PBS. Samples were concentrated by centrifugation at 8000×*g* for 10 min and resuspended in 1 mL of PBS. Genomic DNA was harvested from each sample using the Promega Maxwell 16 Instrument^®^ and the Cell DNA Purification Kit. The tagged region that harbored the 30-bp barcode was amplified in triplicate from genomic DNA using primer P47 and primers P48, P51–73 (Supplementary Table 4). The PCR products were run on a 1.5% agarose gel, pooled, extracted from the gel using Qiagen QIAquick^®^ gel extraction kit and quantified (Invitrogen Quant-iT^™^ dsDNA Assay Kit, High Sensitivity). The purified PCR products were combined in equimolar concentrations and sequenced on an Illumina Miseq instrument (Miseq Reagent Kit v2, 50-cycle, Illumina) using custom sequencing primer P49^11^ (Supplementary Table 4) with a mean cluster density of 8.6 × 10^5^ ± 2.5 × 10^5^. Reaper-15-065 was used to discard sequence reads with low quality (≤Q30) and to trim the sequence following the barcode^56^. The trimmed sequences were clustered with QIIME (version 1.9.1) using pick_otus.py with a sequence similarity threshold of 0.9^57,58^. The resulting estimated *N*_*b*_ was mathematically calculated from the frequency of each barcode as described by Abel et al.^11^ using the method of Krimbas and Tsakas^59^. These *N*_*b*_ values were then compared to experimentally determined founding population sizes (CFU) to generate a calibration curve (Supplementary Fig. 4c). As mathematically derived *N*_*b*_ values underestimated the actual *N*_*b*_ value based on the calibration curve, this curve was used to adjust *N*_*b*_ values obtained from organs harvested from infected mice yielding corrected founding population sizes (*N*_*b*_′). For each *N*_*b*_′ value calculated for every organ in each mouse, 95% confidence intervals are presented (Supplementary Figs. 5d and 6d).

### Barcode distribution, allelic frequency, and skew in PABL012_pool_

Barcodes from 30 independently sequenced aliquots from the PABL012_pool_ inoculum were analyzed as described above. Barcodes present in 29 of 30 aliquots (INOC30) were deemed adequately represented in the PABL012_pool_ inoculum given to mice and were included in the calculation of *N*_*b*_ values^11^ (14 hpi *n* = 4612, 24 hpi *n* = 4208). Barcodes not present in INOC30 were filtered from the barcodes recovered from in vivo experiments. To assess input barcode distribution, the maximum allelic frequency of each barcode present in the inoculum was arrayed on the *x*-axis in descending frequency (Supplementary Figs. 5b and 6b), which revealed that the maximum allelic frequency of any single barcode in the total population at both 14 and 24 hpi was 1.2%. The vast majority of tags (>99%) were present in the population at <0.5% (allelic frequency < 0.005). To assess the impact of this minor input barcode (inoculum) skew on the output barcode (mouse organ) recovery, the maximum input frequency of each barcode present in the inoculum was plotted on the *y*-axis verses the maximum output barcode frequency of the identical barcode from a mouse organ (Supplementary Figs. 5c and 6c). Linear regression with best fit analysis was performed and yielded “goodness of fit” *R*^2^ values of 0.157 and 0.097, respectively, suggesting that input barcode allelic frequency had a poor correlation to output barcode frequency, revealing the stochastic nature of barcode loss in our model (GraphPad Prism v7.0b). Had the allelic frequency of the input barcode dictated the output allelic frequency, we would anticipate an observed an *R*^2^ value closer to 1.

### Animal Infections with PABL012_pool_

An inoculum of barcoded PABL012_pool_ was prepared and injected into mice as described. At 14 and 24 hpi, mice were euthanized and the lungs, liver, spleen, GB, stomach, small intestine, cecum, colon, and feces were harvested, weighed, homogenized in 1 mL of PBS, and plated for CFU counts. For *N*_*b*_′ analysis, 500 µL of samples from the lung, spleen, liver, GB, stomach, small intestine, cecum, colon, feces, and 250 µL of inoculum × 30 were independently spread on 100 × 15 mm Petri dishes containing LB with 5 µg per mL irgasan. Plates were grown at 37 °C overnight and bacteria sequenced for barcode diversity as above. *N*_*b*_ values were calculated as described in the previous section and adjusted using the calibration curve with an R script^11^ to estimate in vivo founding population sizes (*N*_*b*_′). When the number of recovered CFUs was below 100, *N*_*b*_′ determination was not performed. “Genetic distance” between the bacterial populations in two different organs was estimated by comparing the barcode allelic frequencies of the two populations using the Cavalli–Sforza chord distance method^12^ as described by Abel et al.^11^. GR was calculated as (1 − genetic distance).

### Simulated bottlenecks and their impact on GR

The Cavalli–Sforza chord distance method of determining GR is sensitive to narrow population bottlenecks especially when the input population has a disproportionate skew in barcode frequencies (as in PABL012_pool,_ Supplementary Figs. 5b and 6b). Thus, it is possible that the experimental PA populations in the liver, GB and intestinal tract appear to be linked because of their high GR, but actually originated as separate, independent events that stochastically share the same dominant barcodes from the inoculum.

To address whether the input barcode distribution from our experimental inoculum (PABL012_pool_) could produce high-GR populations after independently experiencing the narrowest experimental bottleneck observed in our study (M1–10 GB), we performed in silico bottleneck simulations and compared the GR of those simulated populations. First, to model the input population skew, we distributed reads (totaling the average inoculum read count, *n* = 652,410) to create a simulated inoculum population (INOC_Sim) according to the average frequency of each barcode present in PABL012_pool_ (average of INOC 1–30). Next, we determined the median number of unique barcodes present in our experimental bottleneck (M1–10 GB, *n* = 475). We then randomly chose 475 barcodes from our INOC_Sim population where the odds of choosing any one barcode were proportional to its relative frequency in INOC_Sim. Unique read counts (*n* = 644,750 total) as determined by the average barcode frequency distribution in the GBs were assigned to each selected barcode (e.g., the highest read count was assigned to the first selected barcode and the second highest read count was assigned to the second selected barcode, etc.). Barcodes were only assigned a read count value once and all unselected barcodes were given a read count of zero. In this fashion, 500 unique simulated populations were generated, each having experienced the same bottleneck. The *N*_*b*_′ values (*n* = 500) for each simulated population were determined to ensure a similar distribution of founding population sizes as observed experimentally. GR values were then calculated for each pairwise comparison of the 500 simulated populations (*n* = 124,750 comparisons, Supplementary Fig. 7b–e) as described above. Simulated *N*_*b*_′ values ranged from 1 to 209 and GR values ranged from 0.13 to 0.34 (Moderately Low). Simulations never produced the high-GR values we observed in our animal experiments (Supplementary Fig. 7).

### GR threshold determination

Given that the 30 inoculum samples were from the same original stock of PABL012_pool_ and therefore technically identical with the maximum possible experimentally determined GR, we averaged the GR between inoculum samples (−2 standard deviations) at 14 and 24 hpi to set the threshold for “High” levels of GR at ≥0.88. Given that barcode loss is stochastic and that the dominant barcodes found in individual organs are unique to each mouse, we determined inter-mouse (between mice) GR between organs at 14 and 24 hpi (Supplementary Fig. 4d). We defined “Moderately High” GR by averaging both the inter-mouse (between mice) and intramouse (within a single mouse) GR values which yielded a value of 0.41 (Mod. High, values ≥0.41 and ≤0.88). The populations of PA in organs between mice (inter-mouse) were the least likely to be related (the lowest GR values) so we averaged these least-related organs (intermouse LG, LV, ST, GB, IN, CM, CO, FE at 14 and 24 hpi) to define the “Low” GR threshold as ≤0.17. The inter-mouse spleen GR values were specifically excluded from this calculation because they displayed Moderately High (14 hpi) and Moderately Mow (24 hpi) GR, respectively, likely secondary to the presence of large numbers of diverse barcodes in this organ (Supplementary Fig. 4d).

### Electron microscopy

For transmission electron microscopy (TEM), 6- to 8-week-old female BALB/c mice were infected via tail vein with either PABL012_lux_ or PBS. At defined timepoints post injection, GBs were harvested, fixed for at least 48 h at 4 °C in 0.1 M sodium cacodylate buffer pH 7.3 containing 2% paraformaldehyde and 2.5% glutaraldehyde, and postfixed with 2% osmium tetroxide in unbuffered aqueous solution. Samples were rinsed with distilled water, en bloc stained with 3% uranyl acetate, and rinsed again with distilled water. They were then dehydrated in ascending grades of ethanol, transitioned with propylene oxide, embedded in the resin mixture of Embed 812 kit, and cured in a 60 °C oven. Samples were sectioned on a Leica Ultracut UC6 ultramicrotome. One-micrometer thick sections were collected and stained with Toluidine Blue O. Seventy-nanometer (thin) sections were collected on 200 mesh copper grids and stained with uranyl acetate and Reynolds lead citrate. Images were obtained using the FEI Tecnai Spirit G2 transmission electron microscope with the help of the Northwestern Center for Advanced Microscopy (CAM).

### Histology

For histological sections, 6- to 8-week-old female BALB/c mice were infected via tail vein with either PABL012_lux_, PABL012∆*pscJ*_lux_, or PBS. At defined points postinjection, GBs were harvested and fixed in 4% paraformaldehyde solution for a minimum of 48 h. Samples were then paraffin embedded, processed, and stained with hematoxylin and eosin (H&E) by the Mouse Histology and Phenotyping Laboratory (MHPL) at Northwestern University. GB sections were imaged on the Zeiss Axioscope with a CRI Nuance Camera, located in the Northwestern University CAM.

### Bile growth assays

*P. aeruginosa* PABL012_lux_ or PAO1_lux_ were grown overnight in MINS medium, inoculated 1:8 into fresh medium the following day and regrown to exponential phase. One milliliter of culture was pelleted, resuspended in PBS, and the OD_600_ adjusted to 0.7. This culture was diluted 1:100 and inoculated in triplicate into 180 µL of LB, PBS, MINS medium, or a 35–40% bile solution. To prepare the bile solution, 6- to 12-week-old BALB/c mice were anesthetized and sacrificed by cervical dislocation. GBs were harvested intact and lanced to release bile contents. Gentle centrifugation was used to pellet tissue contents, liberating free bile. Freshly harvested bile was diluted to 35–40% with PBS. Following inoculation, cultures were grown, shaking, at 37 °C for 24 h. Samples were taken at time 0, 4, and 24 h, diluted, and plated to quantify CFU.

### Minimal inhibitory concentrations

Minimal inhibitory concentrations (MICs) for all PA strains used were determined in triplicate using the broth microdilution protocol described by Wiegand et al.^60^ (Supplementary Table 1). The following antibiotics were prepared from commercially available sources and were used to assess MICs: piperacillin/tazobactam (Pip/Tazo), cefepime (Cep), ceftazidime (Ctz), ciprofloxacin (Cipro), meropenem (Mero), gentamicin, colistin (Col), and aztreonam (Az).

### Statistical analysis

Analyses were performed with the help of GraphPad Prism v7.0b. Data are represented as geometric mean (horizontal line or box) ±standard deviation (SD) when multiple mice were challenged. Bacterial CFU is represented as CFU per gram organ and organs from individual mice are represented by a single data point on each graph (circle if CFU recovered, diamond if no CFU recovered). A non-parametric ANOVA test with Kruskal–Wallis correction for multiple comparisons was used to compare bacterial growth in specific media given small samples sizes and non-normal distributions (Supplementary Fig. 1).

## Supplementary information

Supplementary Information

## Data Availability

The data that support the findings of this study are included in the paper and or its Supplementary Information files. Source data for all figures are provided as a separate Source Data file.

## Source Data

Source Data
