# Supplementary material for: Systemic infection facilitates transmission of Pseudomonas aeruginosa in mice
Source: Nat Commun. 2020 Jan 28;11:543. doi: 10.1038/s41467-020-14363-4 (PMC6987207; doi:10.1038/s41467-020-14363-4)
Supplement: Supplementary file 1 — Supplementary Information [file 41467_2020_14363_MOESM1_ESM.pdf]

Systemic infection facilitates transmission of *Pseudomonas aeruginosa* in mice

Bachta, et al.

Supplementary Table 1. *P. aeruginosa* Strain Summary

						MICs (μg/mL)							
**Strain**	**ST Type**	**Source**	**Geography**	**NCBI Accession Number**	***exoU/S***	**Pip/Tazo**	**Cep**	**Ctz**	**Mero**	**Cipro**	**Gent**	**Col**	**Az**
PABL002	253	Bloodstream	Chicago	GCA_003412285.1	U	**2/4**	**1**	**2**	**<0.25**	**<0.25**	**1**	**1**	**4**
PABL012	Unk.	Bloodstream	Chicago	CP031659.1	S	**4/4**	**2**	**2**	**0.5**	**<0.25**	**1**	**1**	**4**
PABL016	2555	Bloodstream	Chicago	GCA_003412165.1	U	**4/4**	**2**	**1**	**<0.25**	**<0.25**	**2**	**4**	**4**
PABL017	2167	Bloodstream	Chicago	CP031660.1	S	**4/4**	**2**	**2**	**0.5**	**<0.25**	**2**	**1**	**4**
PABL028	Unk.	Bloodstream	Chicago	GCA_003411845.1	S	**4/4**	**2**	**2**	**<0.25**	**<0.25**	**4**	**1**	**4**
PABL030	Unk.	Bloodstream	Chicago	GCA_003412115.1	S	**4/4**	**1**	**1**	**0.5**	**<0.25**	**2**	**1**	**8**
PABL041	253	Bloodstream	Chicago	GCA_003411465.1	U	**4/4**	**1**	**1**	**1**	**<0.25**	**1**	**1**	**4**
PABL046	Unk.	Bloodstream	Chicago	GCA_003411535.1	S	**16/4**	**8**	**2**	**8**	**128**	**8**	**1**	**16**
PABL049	244	Bloodstream	Chicago	GCA_003411745.1	S	**8/4**	**4**	**2**	**4**	**<0.25**	**1**	**2**	**16**
PABL095	348	Bloodstream	Chicago	GCA_003410805.1	S	**8/4**	**4**	**2**	**4**	**4**	**1**	**2**	**8**
PABL107	377	Bloodstream	Chicago	GCA_003410475.1	U	**8/4**	**4**	**2**	**4**	**<0.25**	**1**	**2**	**4**
PAC6	1021	Bloodstream	Taiwan		U	**8/4**	**1**	**2**	**1**	**<0.25**	**1**	**1**	**8**
S10	244	Bloodstream	Taiwan		S	**8/4**	**1**	**1**	**1**	**<0.25**	**1**	**1**	**8**
PA01	549	Wound	Australia	NC_002516.2	S	**4/4**	**2**	**2**	**2**	**<0.25**	**2**	**2**	**8**
PA14	253	Wound	Unk.	NC_008463.1	U	**4/4**	**2**	**2**	**2**	**<0.25**	**1**	**1**	**8**

U = exoenzyme U, S = exoenzyme S; Unk. = unknown
Pip/Tazo = piperacillin-tazobactam, Cep = cefepime, Ctz = ceftazidime, Mero = meropenem, Cipro = ciprofloxacin, Gent = gentamicin, Col = Colistin, Az = aztreonam
Resistant, Intermediate: Clinical Laboratory Standards Institute, MIC Interpretive Standards (µg/mL), 2018

Supplementary Table 2. Bacterial strain list

Species	Strain ID	Relevant Characteristics	Reference
E. coli	TOP-10	F- mcrA Δ(mrr-hsdRMS-mcrBC) φ80lacZΔM15 ΔlacX74 nupG recA1 araD139 Δ(ara-leu)7697 galE15 galK rpsL(StrR) endA1 λ-	Invitrogen
E. coli	S17-1 λpir	Sm^R; *pro, thi, hsd*R^-M^+, RP4-2-Tc:Mu;Km:Tn7 λpir	Simon (1983)
E. coli	SM10 λpir	Km^R*; thi*-1 *thr, leu, tonA, lacY, supE, recA*::RP4-2-Tc::Mu λpir	Simon (1983)
P. aeruginosa	PABL002	human bacteremia isolate (presumed source: intraabdominal abscess)	Scheetz (2009)
P. aeruginosa	$PABL002_{lux}$	As above, luciferase cassette cloned into *attB* site	This study
P. aeruginosa	PABL012	human bacteremia isolate (presumed source: uti)	Scheetz (2009)
P. aeruginosa	$PABL012_{lux}$	As above, luciferase cassette cloned into *attB* site	This study
P. aeruginosa	$PABL012_{GM}$	As above, with gentamicin cassette cloned into *attB* site	This study
P. aeruginosa	$PABL012_{lacZ}$	As above, with *lacZ* cloned into *attB* site	This study
P. aeruginosa	PABL012Δ*pscJ*	Clean deletion of *pscJ* (Δ5-245) rendering strain T3S deficient	This study
P. aeruginosa	PABL012Δ$pscJ_{lux}$	As above, with luciferase cassette cloned into *attB* site	This study
P. aeruginosa	PABL016	human bacteremia isolate (presumed source: Indwelling catheter)	Scheetz (2009)
P. aeruginosa	$PABL016_{lux}$	As above, luciferase cassette cloned into *attB* site	This study
P. aeruginosa	PABL017	human bacteremia isolate (presumed source: pneumonia)	Scheetz (2009)
P. aeruginosa	$PABL017_{lux}$	As above, luciferase cassette cloned into *attB* site	This study
P. aeruginosa	PABL028	human bacteremia isolate (presumed source: pneumonia)	Scheetz (2009)
P. aeruginosa	$PABL028_{lux}$	As above, luciferase cassette cloned into *attB* site	This study
P. aeruginosa	PABL030	human bacteremia isolate (presumed source: unknown)	Scheetz (2009)
P. aeruginosa	$PABL030_{lux}$	As above, luciferase cassette cloned into *attB* site	This study
P. aeruginosa	PABL041	human bacteremia isolate (presumed source: unknown)	Scheetz (2009)
P. aeruginosa	$PABL041_{lux}$	As above, luciferase cassette cloned into *attB* site	This study
P. aeruginosa	PABL046	human bacteremia isolate (presumed source: unknown)	Scheetz (2009)
P. aeruginosa	$PABL046_{lux}$	As above, luciferase cassette cloned into *attB* site	This study
P. aeruginosa	PABL049	human bacteremia isolate (presumed source: biliary stent)	Scheetz (2009)
P. aeruginosa	$PABL049_{lux}$	As above, luciferase cassette cloned into *attB* site	This study
P. aeruginosa	PABL095	human bacteremia isolate (presumed source: pneumonia)	Scheetz (2009)
P. aeruginosa	$PABL095_{lux}$	As above, luciferase cassette cloned into *attB* site	This study
P. aeruginosa	PABL107	human bacteremia isolate (presumed source: unknown)	Scheetz (2009)
P. aeruginosa	$PABL107_{lux}$	As above, luciferase cassette cloned into *attB* site	This study
P. aeruginosa	PAC6	human bacteremia isolate (Shanghai Fever)	Chuang (2014)
P. aeruginosa	$PAC6_{lux}$	As above, luciferase cassette cloned into *attB* site	This study
P. aeruginosa	S10	human bacteremia isolate (Shanghai Fever)	Chuang (2014)
P. aeruginosa	$S10_{lux}$	As above, luciferase cassette cloned into *attB* site	This study
P. aeruginosa	PAO1	human wound isolate	Halloway (1955)
P. aeruginosa	$PAO1_{lux}$	As above, luciferase cassette cloned into *attB* site	This study
P. aeruginosa	PA14	human wound isolate	Rahme (1995)
P. aeruginosa	$PA14_{lux}$	As above, luciferase cassette cloned into *attB* site	This Study

Supplementary Table 3. Plasmid list

Plasmid	Relevant Characteristics	Reference
pEXG2	Gm^R; allelic exchange vector with pBR origin, *sacB*+	Rietsch (2005)
pEXG2Δ*pscJ*	Gm^R; allelic exchange vector for making unmarked deletion of *pscJ* (Δ5-245); strains are null for T3 secretion	This study
pFLP2	Ap^R/Cb^R; *oriT*+ *sacB*+, Flp recombinase	Hoang (1998)
pEX18.Gm	Gm^R; *oriT*+ *sacB*+, gene replacement vector with MCS from pUC18	Hoang (1998)
pminiCTX-1	Tc^R; self-proficient integration vector with Ω-*FRT*-*attP*-MCS, *ori, int,* and *oriT*	Hoang (2000)
pminiCTX*npt2lux*	Tc^R; luciferase expressing cassette driven by *pnpt2* promoter ligated into miniCTX-1	Diaz (2008)
pminiCTX-1_{GM}	Tc^R Gm^R; Gentamicin resistance cassette (amplified from pEX18.Gm) driven by native promoter ligated into miniCTX-1	This study
pminiCTX-1_{lacZ}	Tc^R; *lacZ* expressing cassette ligated into miniCTX-1	Gift of S. Lory
pminiCTX$_{STAMP}$	Tc^R Gm^R; barcoded Gentamicin resistance cassette (amplified from pEX18.Gm) driven by native promoter ligated into EcoRI site in miniCTX-1	This study

Supplementary Table 4. Primer list

Primer	Sequence 5'-3'
pscJ – 5-1 HindIII	GCATAAATGTAAAGCAGCAGCAGCGATCCTGC
pscJ - 5-2	*AACTCGAGCCGCAAGCATGCTGAA*CGTTCGCCTCATTGGGTC
pscJ - 3 -1	*TTCAGCATGCTTGCGGCTCGAGTT*CGGCAACGGGGCTGAGCG
pscJ - 3 -2 HindIII	CTAGAGTCGACCTGCAGACTGCTCCTGGTAAACCTCG
pscJ - 5	CCATGGCAATCCCTGGCCGGC
pscJ - 3	CCGGTAGTAGCGATTGCAGCGC
Gm-pCTX_F-BamHI-2	CGGGATCCCGCCGCTCATGAGACAATAACCCTGA
Gm-pCTX_R-EcoRI	CGGAATTCCGAAAGTATATATGAGTAAACTT
P49	ACGCTCTTCCGATCTTGTAAAACGACGGCCAGT
P47	AATGATACGGCGACCACCGAGATCTACACTCTTTCCCTACACGACGCTCTTCCGATCT
P80	TATCGATAAGCTTGATATCGGTAAACTTGGTCTGACAATCGATGC
P110	TGGATCCCCCGGGCTGCAGGCAGGAAACAGCTATGACNNNNNNNNNNNNNNNNNNNNNNNNNNNNNNACTGGCCGTCGTTTTACACGCCGCTCATGAGACAATAACCCTGA
P48	CAAGCAGAAGACGGCATACGAGATCGTGATGTGACTGGAGTTCAGACGTGTGCTCTTCCGATCATTCAGCAGACCTACGATGTCGGGG
P51	CAAGCAGAAGACGGCATACGAGATACATCGGTGACTGGAGTTCAGACGTGTGCTCTTCCGATCATTCAGCAGACCTACGATGTCGGGG
P52	CAAGCAGAAGACGGCATACGAGATGCCTAAGTGACTGGAGTTCAGACGTGTGCTCTTCCGATCATTCAGCAGACCTACGATGTCGGGG
P53	CAAGCAGAAGACGGCATACGAGATTGGTCAGTGACTGGAGTTCAGACGTGTGCTCTTCCGATCATTCAGCAGACCTACGATGTCGGGG
P54	CAAGCAGAAGACGGCATACGAGATCACTGTGTGACTGGAGTTCAGACGTGTGCTCTTCCGATCATTCAGCAGACCTACGATGTCGGGG
P55	CAAGCAGAAGACGGCATACGAGATATTGGCGTGACTGGAGTTCAGACGTGTGCTCTTCCGATCATTCAGCAGACCTACGATGTCGGGG
P56	CAAGCAGAAGACGGCATACGAGATGATCTGGTGACTGGAGTTCAGACGTGTGCTCTTCCGATCATTCAGCAGACCTACGATGTCGGGG
P57	CAAGCAGAAGACGGCATACGAGATTCAAGTGTGACTGGAGTTCAGACGTGTGCTCTTCCGATCATTCAGCAGACCTACGATGTCGGGG
P58	CAAGCAGAAGACGGCATACGAGATCTGATCGTGACTGGAGTTCAGACGTGTGCTCTTCCGATCATTCAGCAGACCTACGATGTCGGGG
P59	CAAGCAGAAGACGGCATACGAGATAAGCTAGTGACTGGAGTTCAGACGTGTGCTCTTCCGATCATTCAGCAGACCTACGATGTCGGGG
P60	CAAGCAGAAGACGGCATACGAGATGTAGCCGTGACTGGAGTTCAGACGTGTGCTCTTCCGATCATTCAGCAGACCTACGATGTCGGGG
P61	CAAGCAGAAGACGGCATACGAGATTACAAGGTGACTGGAGTTCAGACGTGTGCTCTTCCGATCATTCAGCAGACCTACGATGTCGGGG
P62	CAAGCAGAAGACGGCATACGAGATTGTTGACTGTGACTGGAGTTCAGACGTGTGCTCTTCCGATCATTCAGCAGACCTACGATGTCGGGG
P63	CAAGCAGAAGACGGCATACGAGATACGGAACTGTGACTGGAGTTCAGACGTGTGCTCTTCCGATCATTCAGCAGACCTACGATGTCGGGG
P64	CAAGCAGAAGACGGCATACGAGATTCTGACATGTGACTGGAGTTCAGACGTGTGCTCTTCCGATCATTCAGCAGACCTACGATGTCGGGG
P65	CAAGCAGAAGACGGCATACGAGATCGGGACGGGTGACTGGAGTTCAGACGTGTGCTCTTCCGATCATTCAGCAGACCTACGATGTCGGGG
P66	CAAGCAGAAGACGGCATACGAGATGTGCGGACGTGACTGGAGTTCAGACGTGTGCTCTTCCGATCATTCAGCAGACCTACGATGTCGGGG
P67	CAAGCAGAAGACGGCATACGAGATCGTTTCACGTGACTGGAGTTCAGACGTGTGCTCTTCCGATCATTCAGCAGACCTACGATGTCGGGG
P68	CAAGCAGAAGACGGCATACGAGATAAGGCCACGTGACTGGAGTTCAGACGTGTGCTCTTCCGATCATTCAGCAGACCTACGATGTCGGGG
P69	CAAGCAGAAGACGGCATACGAGATTCCGAAACGTGACTGGAGTTCAGACGTGTGCTCTTCCGATCATTCAGCAGACCTACGATGTCGGGG
P70	CAAGCAGAAGACGGCATACGAGATTACGTACGGTGACTGGAGTTCAGACGTGTGCTCTTCCGATCATTCAGCAGACCTACGATGTCGGGG
P71	CAAGCAGAAGACGGCATACGAGATATCCACTCGTGACTGGAGTTCAGACGTGTGCTCTTCCGATCATTCAGCAGACCTACGATGTCGGGG
P72	CAAGCAGAAGACGGCATACGAGATATATCAGTGTGACTGGAGTTCAGACGTGTGCTCTTCCGATCATTCAGCAGACCTACGATGTCGGGG
P73	CAAGCAGAAGACGGCATACGAGATAAAGGAATGTGACTGGAGTTCAGACGTGTGCTCTTCCGATCATTCAGCAGACCTACGATGTCGGGG

Supplementary Figure 1

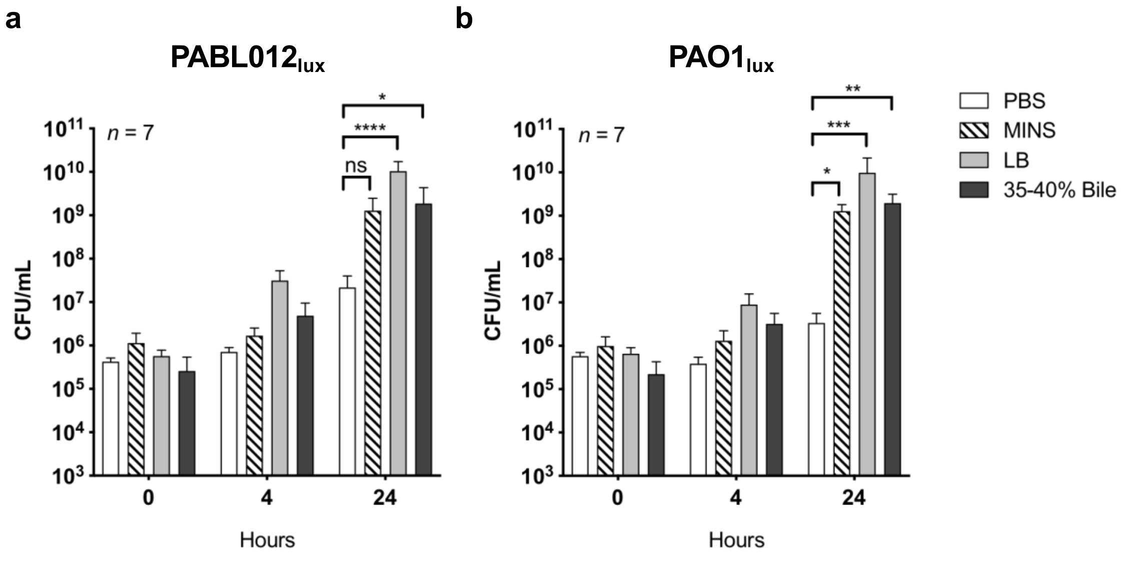

Bile promotes the replication of *P. aeruginosa*. In vitro bacterial growth was enumerated for PABL012$_{lux}$ (**a**) and PAO1$_{lux}$ (**b**) in PBS, MINS, LB, or PBS supplemented with 35-40% mouse bile at 0, 4, and 24-hour post-inoculation. Columns represent the mean CFU per mL with standard deviation (SD, whiskers) (*n* = 7 total, 2 replicates). *p*-values were calculated via a Kruskal-Wallis analysis to correct for multiple comparisons. ns (not significant), (*) $p < 0.05$, (**) $p < 0.005$, (***) $p < 0.001$, (****) $p < 0.0001$.

Supplementary Figure 2

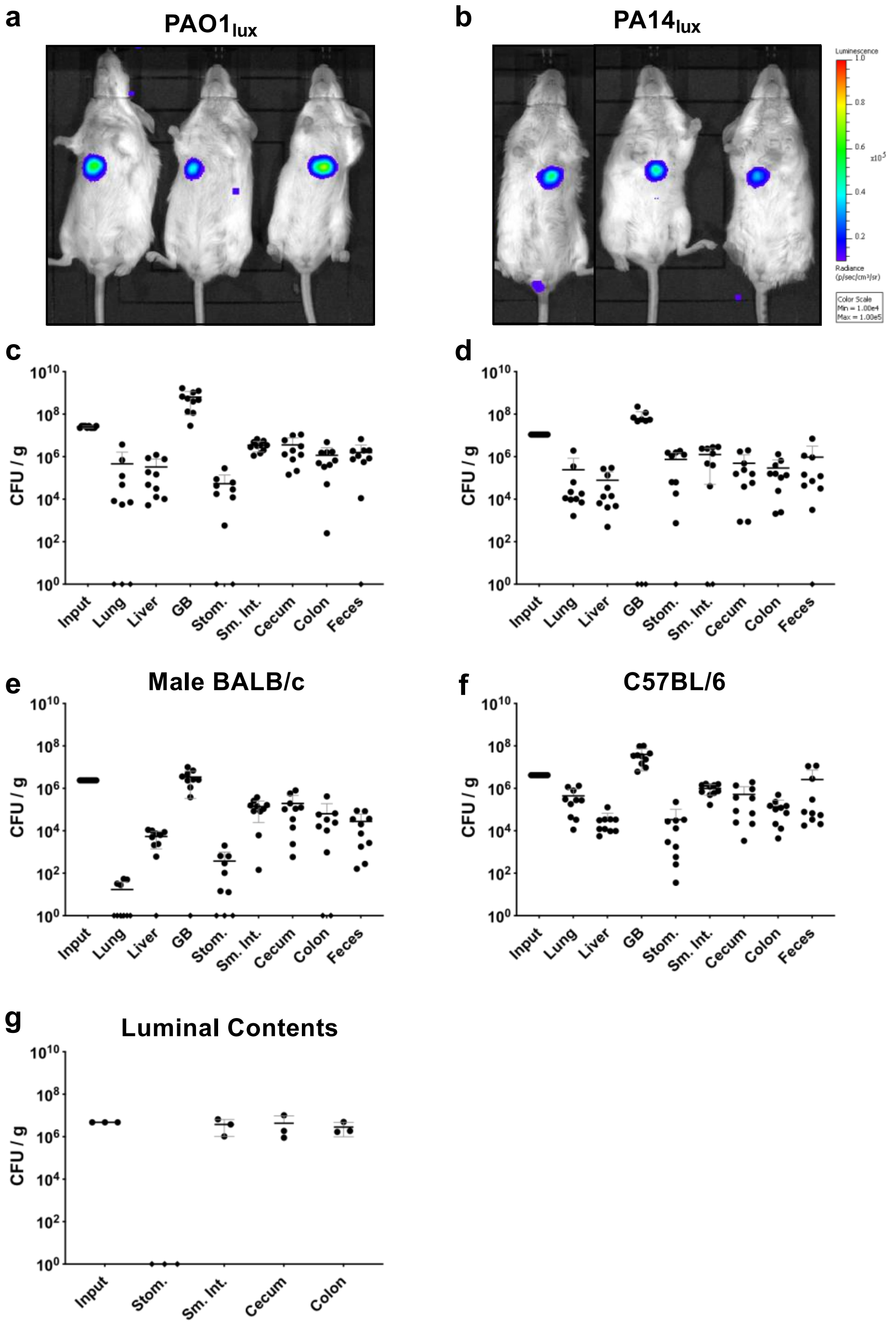

Gastrointestinal shedding of *P. aeruginosa* is universal and independent of mouse genetics or gender. BALB/c mice were infected intravenously with ~1 x 10^6 CFU of PAO1$_{lux}$ (**a** *n* = 5 x 2 replicates) and ~2 x 10^5 CFU of PA14$_{lux}$ (**b** *n* = 6 x 2 replicates) and imaged on the IVIS at 24 hpi. Mice were subsequently euthanized, and bacteria were enumerated from several anatomical sites. **c, d** Bacterial density as measured by CFU per gram

organ [CFU/g] are presented (black circles). **e** Age-matched male BALB/c mice (*n* = 5 x 2 replicates) were infected intravenously with ~2 x 10^6 CFU of $PABL012_{lux}$, organs harvested as 24 hpi and plated for CFU enumeration. **f** Age-matched C57BL/6 (*n* = 5 x 2 replicates) mice were infected intravenously with ~2 x 10^6 CFU of $PABL012_{lux}$, organs harvested as 24 hpi and plated for CFU enumeration. **g** BALB/c mice (*n* = 3) were infected intravenously with ~2 x 10^6 CFU of $PABL012_{lux}$, bacteria were enumerated from the luminal contents of the GI tract by extrusion of contents following dissection. Each symbol represents one mouse; when no bacteria were recovered, mice are represented as diamonds on the *x*-axis. Geometric means (horizontal lines) and SD (whiskers) are shown. GB (gallbladder), Stom. (stomach), Sm. Int. (small intestine).

Supplementary Figure 3

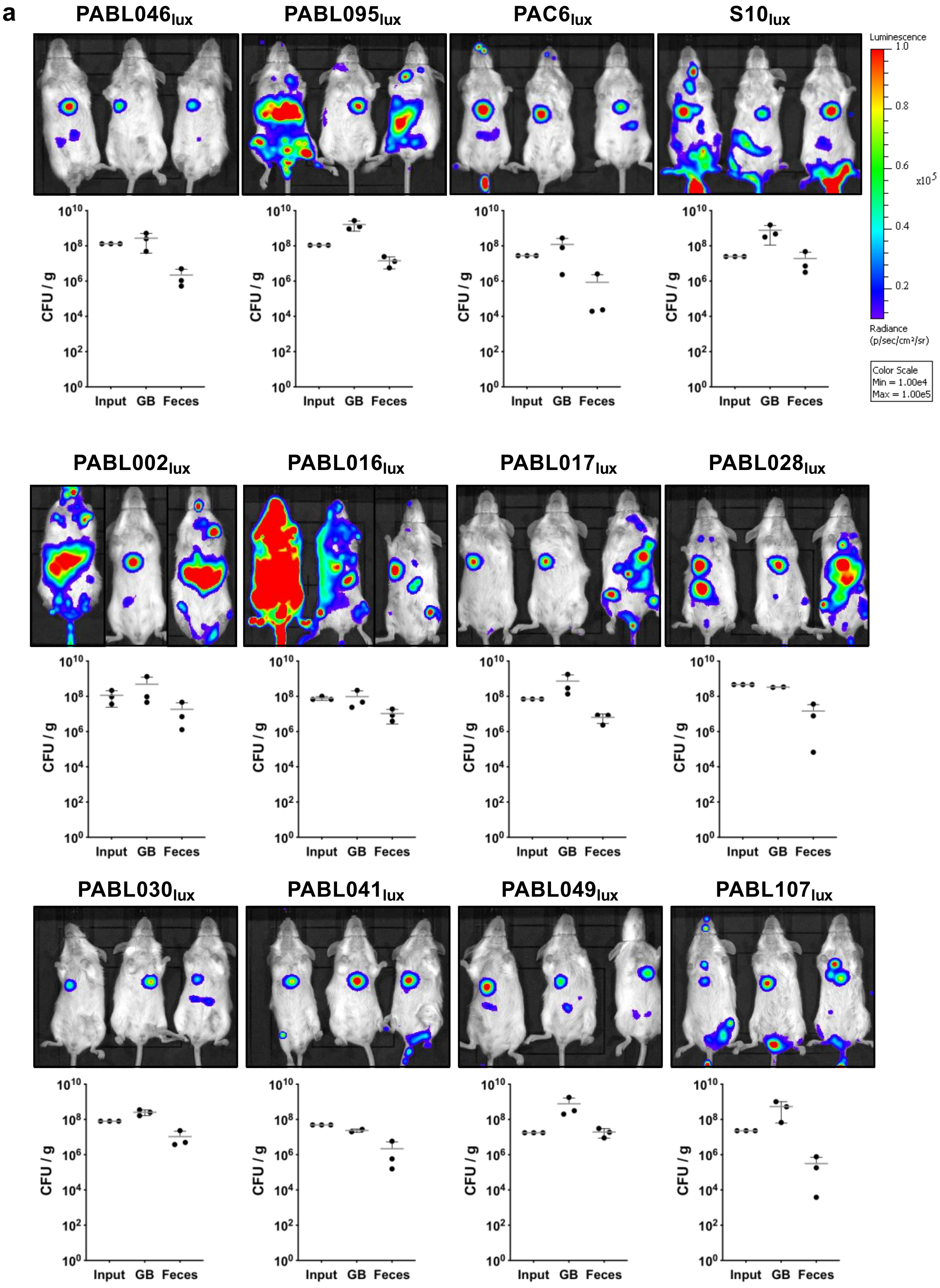

b

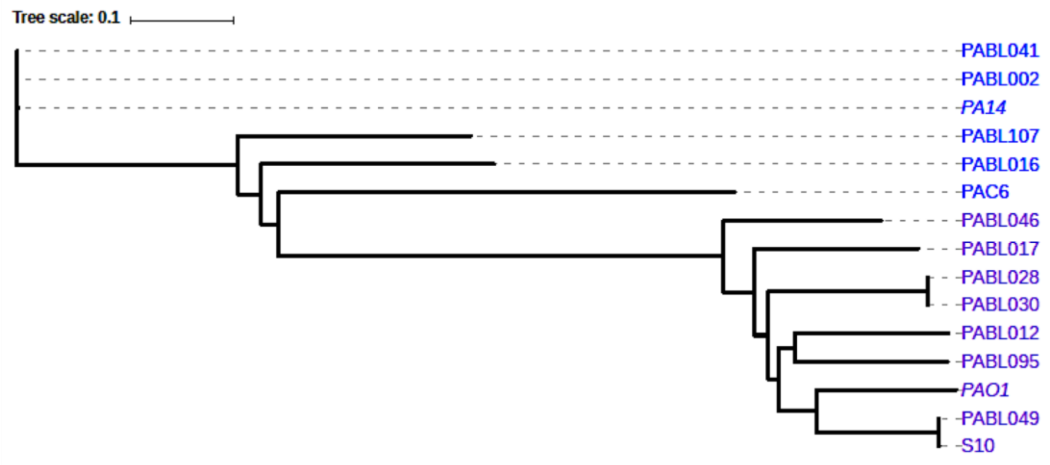

Dissemination to the gallbladder and fecal excretion are shared features among diverse clinical isolates of *P. aeruginosa*. a BALB/c mice were infected intravenously with 8.8 x 10^5 – 4.8 x 10^7 (dose specific to each strain, representative replicate) of *P. aeruginosa* strains $PABL046_{lux}$, $PABL095_{lux}$, $PAC6_{lux}$, $S10_{lux}$, $PABL002_{lux}$, $PABL016_{lux}$, $PABL017_{lux}$, $PABL028_{lux}$, $PABL030_{lux}$, $PABL041_{lux}$, $PABL049_{lux}$, and $PABL107_{lux}$ and imaged at 24 hpi using IVIS. Mice were subsequently euthanized, and bacteria were enumerated from gallbladder and feces. CFU per gram organ (CFU/g, black circles) of recovered bacteria are presented. Each circle represents one mouse (*n* = 3, minimum 2 replicates). Geometric means (horizontal lines) and SD (whiskers) are shown. **b** A parsimony tree based on SNP loci present in 95% of the genomes was generated using kSNP v3.0.21 and visualized in iTOL. The midpoint rooted tree was used to visualize *exoU*+ strains (blue) and *exoS*+ strains (purple).

Supplementary Figure 4

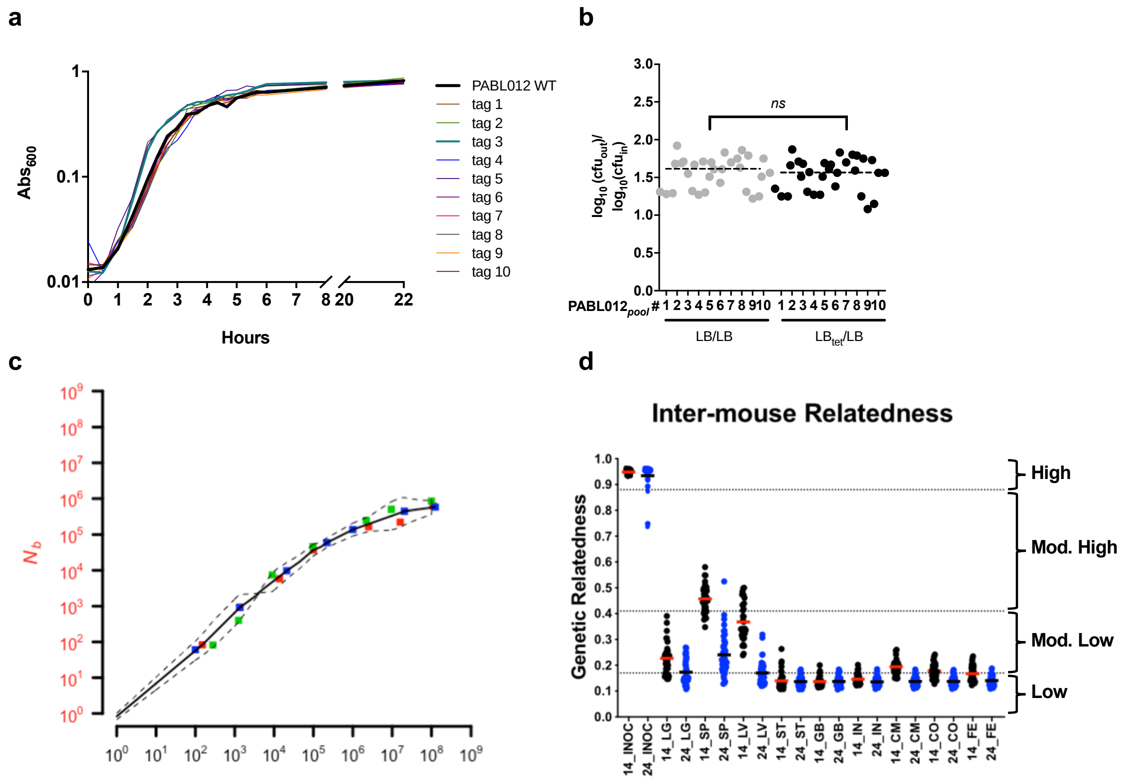

STAMP control experiments and calculations. a In vitro growth of parental PABL012 and a selection of 10 individually barcoded $PABL012_{pool}$ (tag 1-10) strains in liquid culture over 22 hours. **b** The stability of barcode insertion was determined by comparing the output and input CFU for the same 10 individually barcoded strains grown initially in liquid culture without selection for the barcodes (LB) for 20 hours followed by growth on agar plates with tetracycline supplementation for growth of barcoded bacteria (LB_{tet}) or without tetracycline supplementation for growth of both tagged and untagged bacteria (LB). The data are presented as a ratio of output to input CFU (grey circle [LB vs. LB], black circle [LB_{tet} vs. LB]). The dashed line represents the overall median for each condition where $PABL012_{pool}$ strains with tags #1-10 were tested in triplicate (each data point represents one replicate). No significant difference in the ratio of output to input with or without tag selection was detected (p = 0.62, Mann-Whitney test). **c** Calibration curve for correspondence of mathematically derived founding population sizes (N_b, y-axis) with experimentally determined founding population sizes (CFU, x-axis) for three independent technical replicates (filled red, blue, green squares). The solid line indicates the median and the dashed black lines represent the 95% confidence intervals (CI). **d** Genetic relatedness (GR) of the

inoculums and bacteria from the same organ type in different mice (inter-mouse) are presented. For the inoculums (INOC), each circle represents the allelic frequency of barcodes in each inoculum compared to the allelic frequencies of the same barcodes in a different inoculum (technical replicates, n = 30). For inter-mouse GR, each circle represents one organ in pairwise comparison with the same organ from a different mouse (14 hpi black, 24 hpi blue). Geometric means (14 hpi [red] and 24 hpi [black], horizontal bars) are shown. Dashed lines denote GR categories which are defined as High (≥0.88), Moderately High (Mod. High, <0.88, ≥0.41), Moderately Low (Mod. Low, <0.41, ≥0.17), and Low (<0.17). LG (lung), SP (spleen), LV (liver), ST (stomach), GB (gallbladder), IN (small intestine), CM (cecum), CO (colon), FE (feces).

Supplementary Figure 5

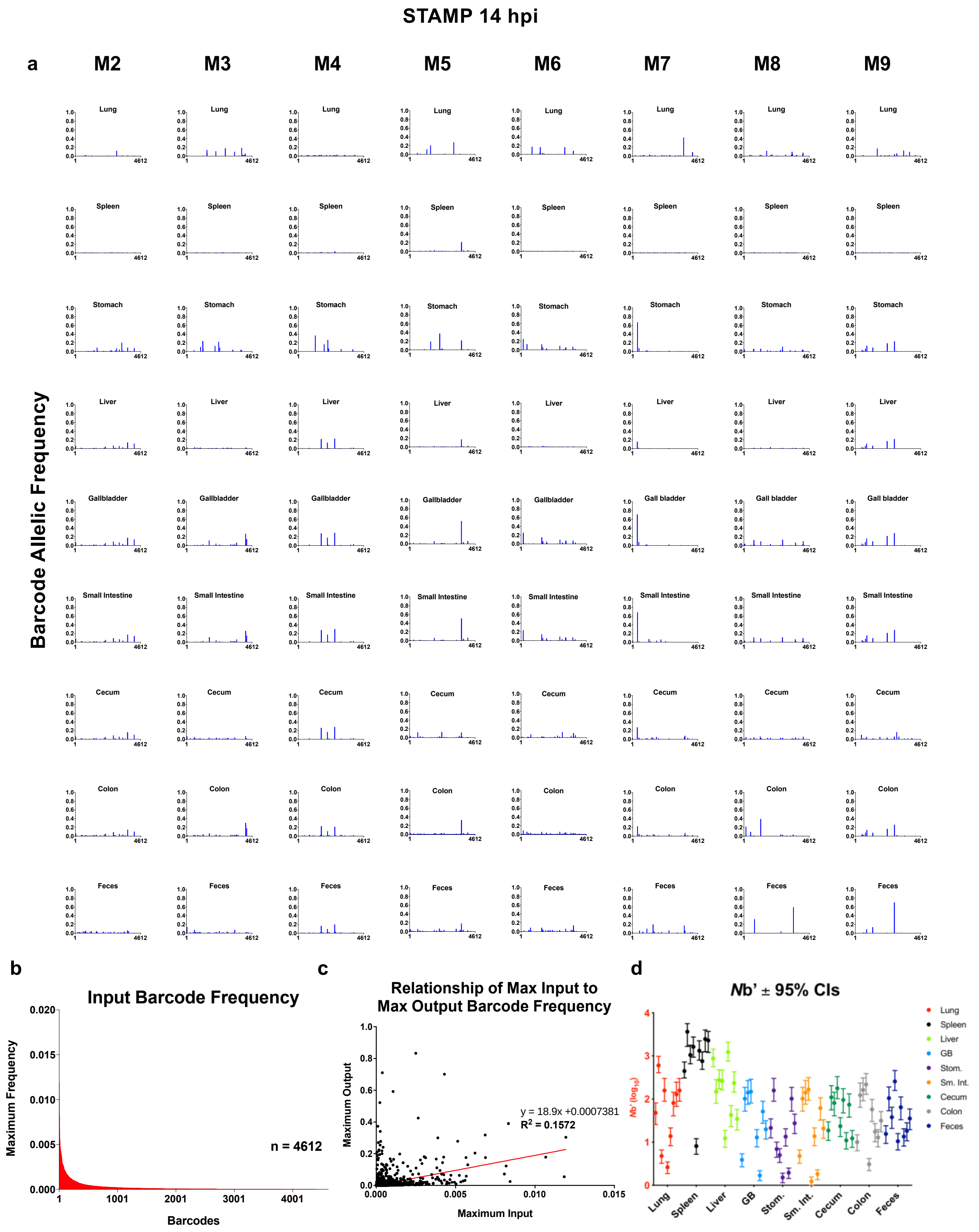

Individual STAMP barcode distributions across all organs in all mice at 14 hpi. BALB/c mice (14 hpi, Mouse 2-9) were infected with *P. aeruginosa* strain PABL012$_{lux}$ by intravenous inoculation of ~2 x 10^6 CFU and organs harvested. **a** Allelic frequencies of all unique barcodes are presented (y-axis) for every organ and every mouse (M2-9, M1 is shown in main text). All 4612 unique barcodes are arranged in identical order along the x-axis. **b** For all 4612 barcodes processed, the maximum frequency found in any inoculum (across all 30 technical replicates) is presented along the y-axis. Barcodes are arranged from most frequent (0.0119, 1.19%) to least frequent (0.000007, 0.0007%). **c** To determine the relationship between input frequency and output frequency for all 4612 barcodes, we plotted the maximum output frequency of that barcode (y-axis) in any mouse versus the maximum input frequency of that barcode found in the inoculum (x-axis). A linear regression was performed, revealing a poor correlation between the input barcode and the output barcode frequency (R^2 = 0.1572). **d** The calculated N_b' values and their associated 95% confidence intervals (CI, whiskers) for every organ in every mouse at 14 hpi; circles, lung (red), spleen (black), liver (light green), gallbladder (GB, light blue), stomach (Stom., purple), small Intestine (Sm. Int, orange), cecum (green), colon (grey), feces (navy).

Supplementary Figure 6

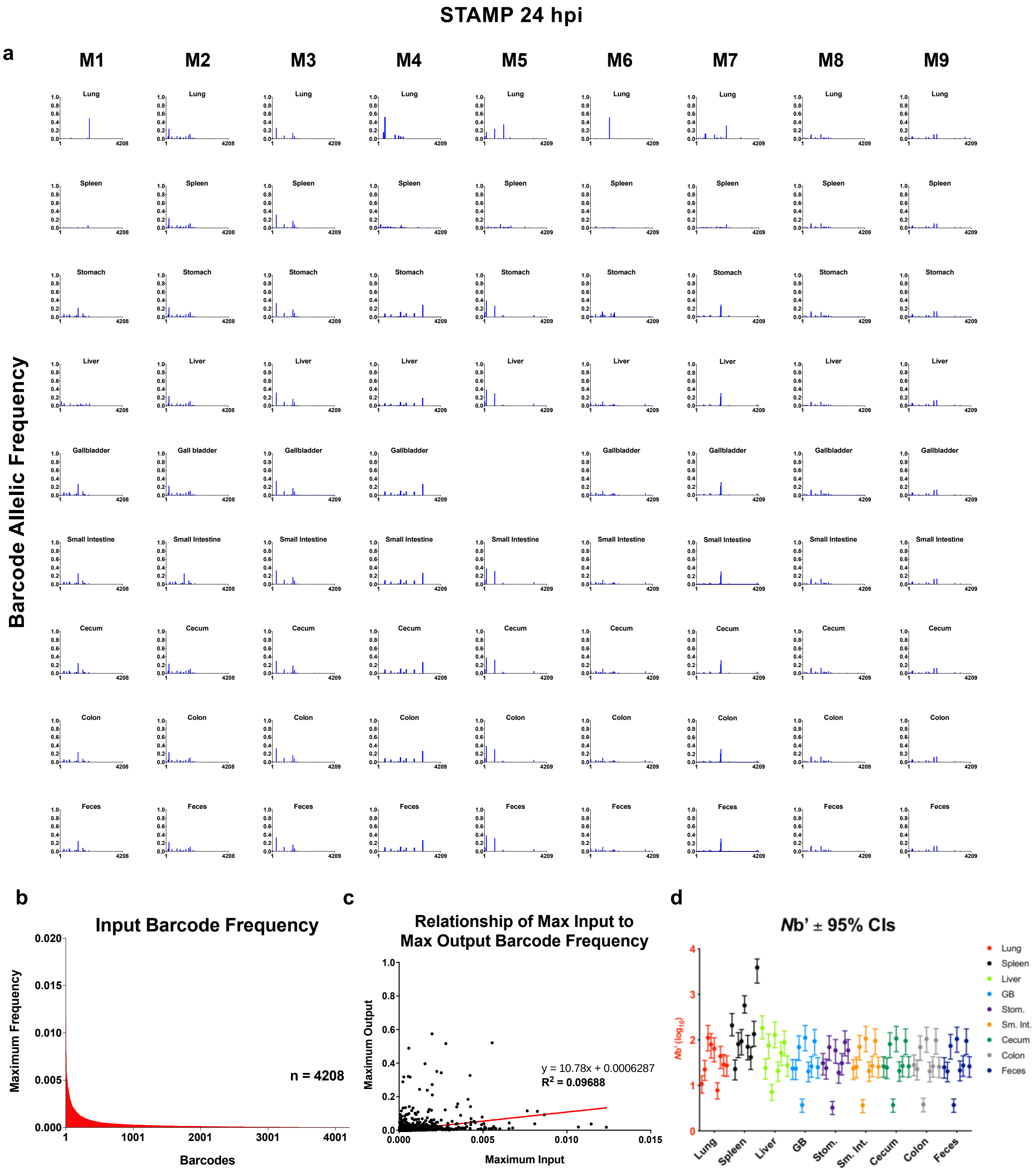

Individual STAMP barcode distributions across all organs in all mice at 24 hpi. BALB/c mice (24 hpi, Mouse 1-9) were infected with *P. aeruginosa* strain $PABL012_{lux}$ by intravenous inoculation of ~2 x 10^6 CFU and organs harvested. **a** Allelic frequencies of all unique barcodes are presented (y-axis) for every organ and every mouse (M1-9, M10 shown in main text). All 4208 barcodes are arranged in identical order along the x-axis. Mouse 5's gallbladder was unable to be processed following dissection. **b** For all 4208 barcodes processed, the maximum frequency found in any inoculum (across all 30 technical replicates) is presented along the y-axis. Barcodes are arranged from most-frequent (0.0124, 1.24%) to least frequent (0.000011, 0.00011%). **c** In order to determine the relationship between input frequency and output frequency for all 4208 barcodes, we plotted the maximum output frequency of that barcode (y-axis) in any mouse versus the maximum input frequency of that barcode found in the inoculum (x-axis). A linear regression was performed, revealing a poor correlation between the input barcode and the output barcode frequency (R^2 = 0.09688). (d) The calculated N_b´ values and their associated 95% confidence intervals (CI, whiskers) for every organ in every mouse at 14 hpi; circles, lung (red), spleen (black), liver (light green), gallbladder (GB, light blue), stomach (Stom., purple), small intestine (Sm. Int, orange), cecum (green), colon (grey), feces (navy).

Supplemental Figure 7

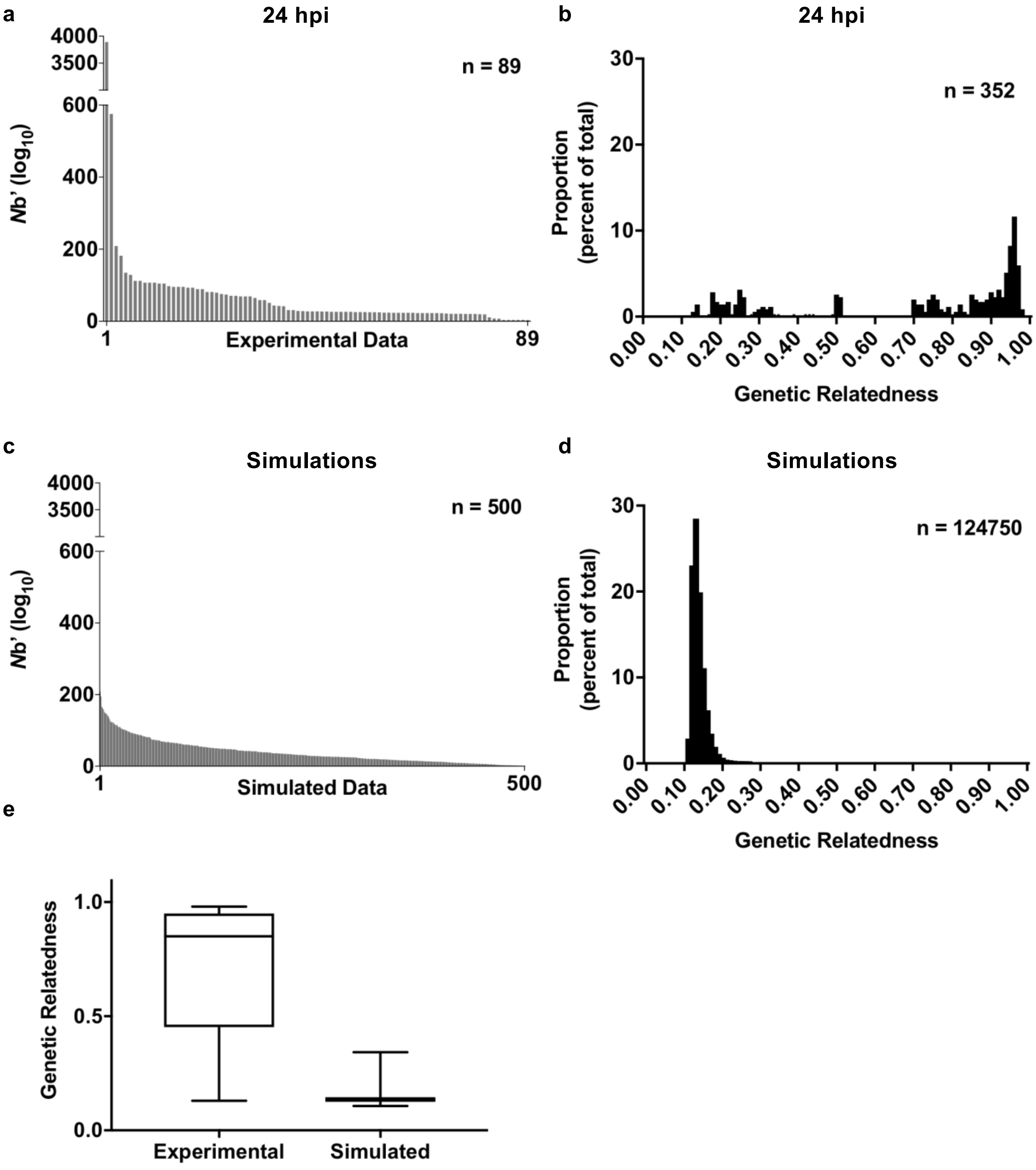

Comparison of experimentally derived and simulated STAMP bottlenecks and genetic relatedness.

a The experimentally determined N_b' values (y-axis) of PA populations sampled from all mouse organs (M1-10) at 24 hpi are sorted along the x-axis from highest to lowest N_b' value (n = 89, max = 3890, min = 3). (b) The genetic relatedness (GR) of these sampled PA populations within each mouse was calculated in a pairwise manner as described in the text (min = 0.13, max 0.98). GR values were rounded to the nearest hundredth,

grouped in 0.01 increments and plotted as a histogram. The y-axis represents the proportion of comparisons producing that GR value (*n* = 352 total comparisons). **c** Using the experimentally determined inoculum barcode distribution and the average frequency distribution of barcodes in the tightest organ bottleneck (Gallbladder at 24hpi), we randomly simulated 500 independent bottleneck events. We calculated $N_b´$ values for all 500 simulated bottleneck events and sorted them along the x-axis highest to lowest $N_b´$ value (max = 209, min = 1). **d** The GR between each of the simulated populations from panel **c** was calculated in a pairwise manner (min = 0.11, max 0.34). GR values were rounded to the nearest hundredth, grouped in 0.01 increments and plotted as a histogram. The y-axis represents the proportion of comparisons producing that GR value (*n* = 124,750 total comparisons). **e** The experimental (**b**) and simulated (**d**) GR values are displayed as a box plot where the median (black line), interquartile range (box), and minimum and maximum (whiskers) are presented. No outliers are present as the whiskers represent maximum and minimum values.
